# The EMT factor ZEB1 paradoxically inhibits EMT in *BRAF*-mutant carcinomas

**DOI:** 10.1172/jci.insight.164629

**Published:** 2023-10-23

**Authors:** Ester Sánchez-Tilló, Leire Pedrosa, Ingrid Vila, Yongxu Chen, Balázs Győrffy, Lidia Sánchez-Moral, Laura Siles, Juan J. Lozano, Anna Esteve-Codina, Douglas S. Darling, Miriam Cuatrecasas, Antoni Castells, Joan Maurel, Antonio Postigo

**Affiliations:** 1 Group of Gene Regulation in Stem Cells, Cell Plasticity, Differentiation, and Cancer, Department of Oncology and Hematology, Institut d’Investigacions Biomèdiques August Pi i Sunyer (IDIBAPS), Barcelona, Spain.; 2 Group of Gastrointestinal and Pancreatic Oncology, Department of Liver, Digestive System and Metabolism, IDIBAPS, Barcelona, Spain.; 3 Biomedical Research Network in Gastrointestinal and Liver Diseases (CIBEREHD), Carlos III National Health Institute (ISCIII), Barcelona, Spain.; 4 Group of Translational Genomics and Targeted Therapeutics in Solid Tumors, IDIBAPS, and Department of Medical Oncology, Hospital Clinic, Barcelona, Spain.; 5 Cancer Biomarker Research Group, Research Centre for Natural Sciences (TKK), and Department of Bioinformatics and 2nd Department of Pediatrics, Semmelweis University, Budapest, Hungary.; 6 Bioinformatics Platform, CIBEREHD, ISCIII, Barcelona, Spain.; 7 National Centre for Genomic Analysis (CNAG) Center for Genomic Regulation (CRG), The Barcelona Institute of Science and Technology (BIST), Barcelona, Spain; 8 Department of Medicine and Health Sciences, Universitat Pompeu Fabra (UPF), Barcelona, Spain.; 9 Department of Oral Immunology, and Center for Genetics and Molecular Medicine, University of Louisville, Louisville, Kentucky, USA.; 10 Group of Molecular Pathology of Inflammatory Conditions and Solid Tumours, Department of Oncology and Hematology, IDIBAPS, Barcelona, Spain.; 11 Department of Pathology, Hospital Clínic and University of Barcelona School of Medicine, Barcelona, Spain.; 12 Department of Gastroenterology, Hospital Clinic and University of Barcelona School of Medicine, Barcelona, Spain.; 13 Molecular Targets Program, Department of Medicine, J.G. Brown Cancer Center, Louisville, Kentucky, USA.; 14 Catalan Institution for Research and Advanced Studies (ICREA), Barcelona, Spain.

**Keywords:** Gastroenterology, Oncology, Colorectal cancer

## Abstract

Despite being in the same pathway, mutations of *KRAS* and *BRAF* in colorectal carcinomas (CRCs) determine distinct progression courses. ZEB1 induces an epithelial-to-mesenchymal transition (EMT) and is associated with worse progression in most carcinomas. Using samples from patients with CRC, mouse models of *Kras*^G12D^ and *Braf*^V600E^ CRC, and a *Zeb1*-deficient mouse, we show that ZEB1 had opposite functions in *KRAS-* and *BRAF-*mutant CRCs. In *Kras*^G12D^ CRCs, ZEB1 was correlated with a worse prognosis and a higher number of larger and undifferentiated (mesenchymal or EMT-like) tumors. Surprisingly, in *Braf*^V600E^ CRC, ZEB1 was associated with better prognosis; fewer, smaller, and more differentiated (reduced EMT) primary tumors; and fewer metastases. ZEB1 was positively correlated in *KRAS-*mutant CRC cells and negatively in *BRAF-*mutant CRC cells with gene signatures for EMT, cell proliferation and survival, and ERK signaling. On a mechanistic level, *ZEB1* knockdown in *KRAS-*mutant CRC cells increased apoptosis and reduced clonogenicity and anchorage-independent growth; the reverse occurred in *BRAF*^V600E^ CRC cells. ZEB1 is associated with better prognosis and reduced EMT signature in patients harboring *BRAF* CRCs. These data suggest that ZEB1 can function as a tumor suppressor in *BRAF-*mutant CRCs, highlighting the importance of considering the *KRAS/BRAF* mutational background of CRCs in therapeutic strategies targeting ZEB1/EMT.

## Graphical abstract

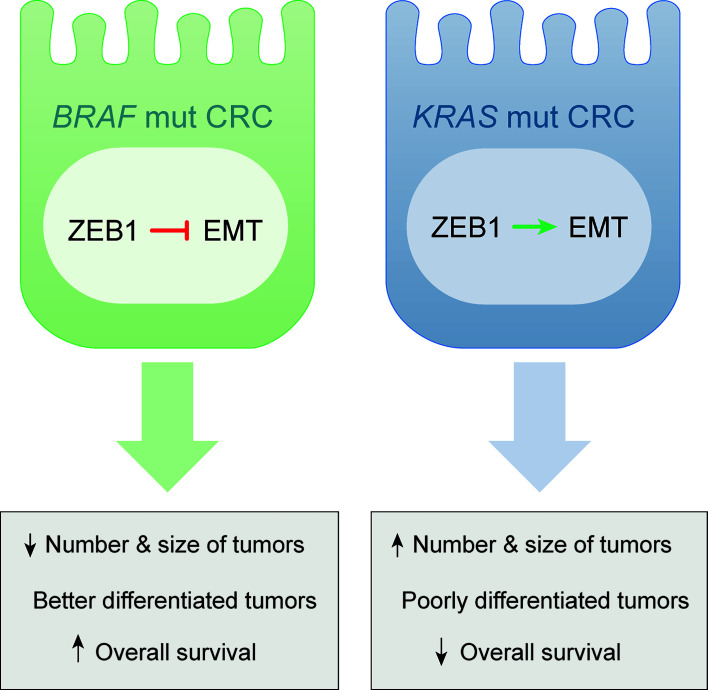

## Introduction

Genetic alterations in the RAS/MAPK/ERK pathway (*ERBB2, ERBB3, NRAS, KRAS, BRAF*) occur in 59% of nonhypermutated CRCs (43% *KRAS*, 3% *BRAF*) and 80% of hypermutated CRCs (30% *KRAS*, 47% *BRAF*) (1). *KRAS* and *BRAF* mutations are most often mutually exclusive and determine differential biological properties (2). Gene expression analyses revealed some differences in the associated gene signatures of mutant *KRAS* and *BRAF* CRCs (3–10). Nonetheless, the underlying molecular mechanisms by which mutant *KRAS* and *BRAF* determine these differences in CRCs are not fully understood.

In addition, more than 90% of all sporadic CRCs exhibit aberrant activation of the Wnt pathway, chiefly through gain-of-function mutations of the *APC* gene, nuclear translocation of β-catenin and β-catenin/TCF4-mediated transcriptional reprogramming of epithelial cells toward more mesenchymal, tumorigenic, stem-like, and pro-invasive or metastatic gene expression signatures (11, 12). This epithelial-to-mesenchymal transition (EMT) is a continuum of phenotypes orchestrated by cell plasticity transcription factors of the ZEB, Snail, and Twist families (13–19). Tumors with a differentiated mesenchymal (EMT-like) phenotype or a hybrid epithelial and mesenchymal state tend to have a poorer prognosis than those with a more epithelial-like phenotype (13–22). Intermediate states displaying some mesenchymal and some epithelial characteristics are particularly important in regulating stemness and tumor initiation capacity (17, 18, 23). Different therapeutic compounds are being tested to target the expression and/or function of these EMT transcription factors for the treatment of different carcinomas, including CRCs (24).

*RAS* synergizes with Wnt signaling to promote progression in CRCs (25, 26). ZEB1 is induced by and synergizes with the Wnt pathway in CRC activating or repressing target genes depending on cell status and/or promoter (27–29). ZEB1 is also downstream of *RAS* and *BRAF* in lung carcinomas and melanomas and mediates some of the signaling of oncogenic *RAS* in lung carcinomas (15, 30–33). ZEB1 associates with poorer survival in most carcinomas, including CRCs (13, 15, 16, 29,34, 35); however, the role of ZEB1 in *BRAF*-mutant CRCs or a potential differential role of ZEB1 in CRCs based on the mutational status have not been explored.

Using human samples of primary CRC with *BRAF* mutations, CRC-established cell lines, transgenic mouse models for *Kras*^G12D^ and *Braf*^V600E^ intestinal tumors, and a *Zeb1*-deficient mouse, we found that ZEB1 is a tumor-promoting factor and induces an EMT phenotype in mouse *Kras*-mutant CRCs but, surprisingly, ZEB1 inhibits the EMT reprogramming of cancer cells and functions as a tumor suppressor in *Braf*-mutant CRCs. ZEB1 determines a better prognosis in patients harboring oncogenic *BRAF* metastatic CRC (mCRC). Our results show that ZEB1 functions as a tumor suppressor in *BRAF*-mutant CRCs, highlighting the need to assess the mutational background of CRC before using therapies that inhibit the expression and/or function of ZEB1.

## Results

### ZEB1 paradoxically determines better survival in Braf-mutant CRCs.

**Figure 1:**
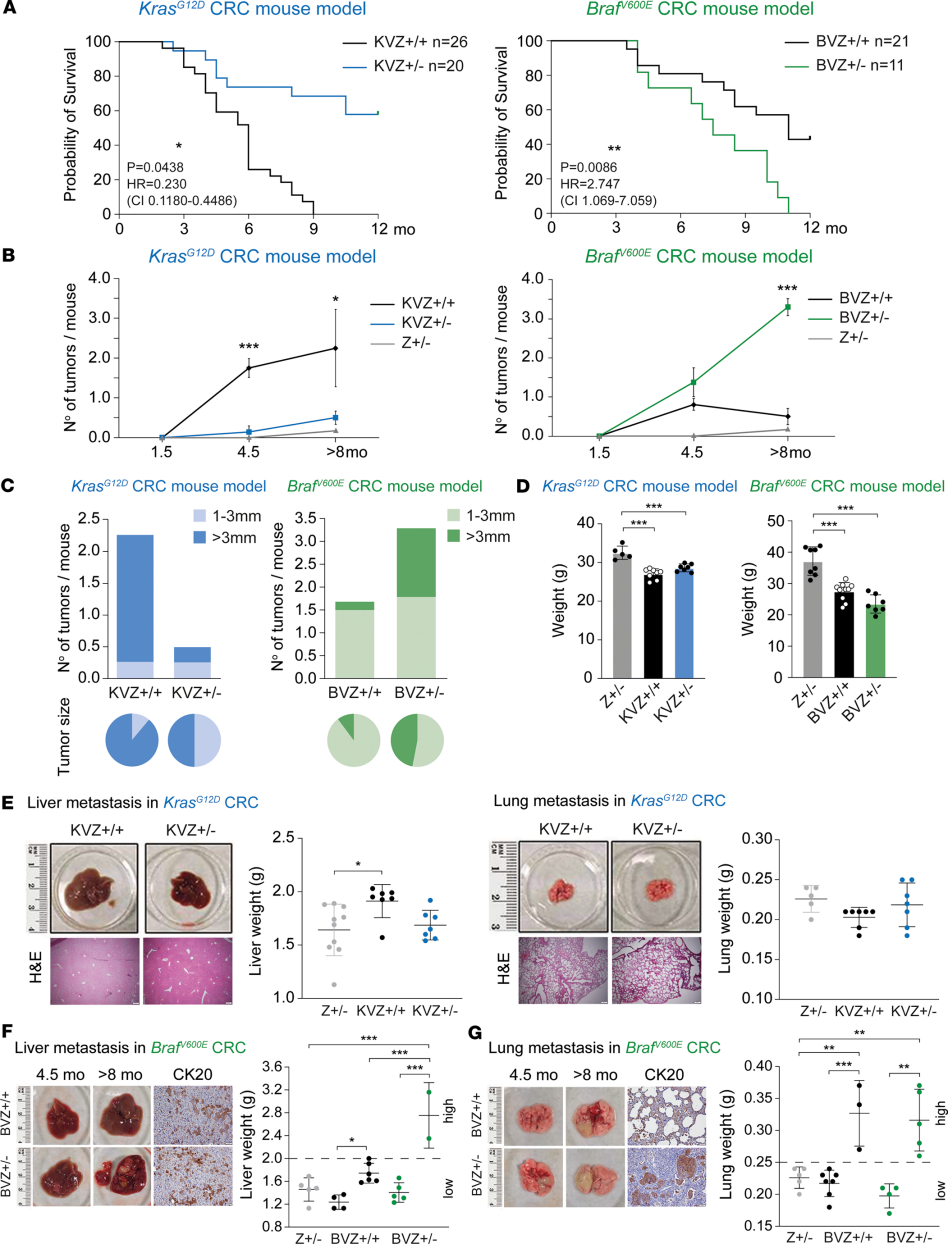
Unlike in *Kras*^G12D^ CRC*, Zeb1* determines a longer survival, smaller and fewer lesions or tumors and metastasis in *Braf^V600E^* CRC. (**A**) *Left:* Overall survival of *Kras^G12D^* mice with 2 (KVZ^+/+^; represented by black line in graph; 12 males, 14 females) or 1 (KVZ^+/–^; blue line in graph; 11 censored; 8 males, 12 females) WT *Zeb1* alleles. *Right:* As in the left panel but for *Braf* mice: BVZ^+/+^; black line; 9 censored; 11 males, 10 females; and BVZ^+/–^; green line; 7 males, 4 females. Log-rank test statistics were applied. (**B**) *Left*: Respective numbers of colonic lesions or tumors of KVZ^+/+^ (represented by black line in graph; *n* = 8, 9, 7 mice), KVZ^+/–^ (blue line; *n* = 5, 9, 7), and Z^+/–^ (gray line; *n* = 6, 6, 11). *Right*: As for BVZ^+/+^ (black lines; *n* = 6, 10, 10), BVZ^+/–^ (green line; *n* = 6, 12, 9). (**C**) Number of macroscopic colonic lesions in *KVZ^+/+^* and *KVZ^+/–^* (left) and *BVZ^+/+^* and *BVZ^+/–^* (right) mice (aged ≥ 8 months) according to tumor size. The pie charts at the bottom represent tumor size independent of the number of tumors. Two-tailed *t* test statistics were used in **B** and **C**. (**D**) No effect on total BW in CRC mouse models: in 4.5-month-old Z^+/–^ (*n* = 5), KVZ^+/+^ (*n* = 8), and KVZ^+/–^ (*n* = 7) mice (left) and in 8-month-old Z^+/–^ (*n* = 8), BVZ^+/+^ (*n* = 10), and BVZ^+/–^ (*n* = 7) mice (right). (**E**) *Kras*^G12D^ mice did not develop metastasis. Images of liver (left) and lung (right) of KVZ^+/+^ and KVZ^+/–^ mice at age >8 months. Respective sample numbers are ZEB1^+/–^ (*n* = 10, 5), KVZ^+/+^ (*n* = 7, 7), and KVZ^+/–^ (*n* = 7, 7) mice. Scale bar: 200 μm. (**F**) *Left*: Liver images of BVZ^+/+^ and BVZ^+/–^ mice and CK20 staining. Scale bar: 50 μm. *Right*: Liver weight in ≥8-month-old Z^+/–^ (*n* = 5), BVZ^+/+^ (*n* = 10), and BVZ^+/–^ (*n* = 7) mice. Stratification of cohorts based on weight above or below the median. (**G**) As in **F**, but for lung in 8-month-old Z^+/–^ (*n* = 5), BVZ^+/+^ (*n* = 10), and BVZ^+/–^ (*n* = 9) mice. Tukey’s multiple comparison test was used in **D**–**G**. *P* values are reported in Supplemental Table 16. ***P ≤ 0.001, **P ≤ 0.01, or *P ≤ 0.05.

Mutations of *KRAS* and *BRAF* in CRC associate with distinct clinical outcomes (3–5). ZEB1 determines a poorer survival in patients with CRC (29), but it remains to be explored whether its protumoral functions are similar in *KRAS-* and *BRAF*-mutant CRCs. There are well-established mouse models of intestinal tumorigenesis harboring mutations of either *Kras* (36) or *Braf* (37, 38) specifically in their intestinal epithelial cells through a *Villin1*-specific Cre (*Kras*^LSL-G12D^; *Vil1*^Cre^ and *Braf*^LSL-V600E^; *Vil1*^Cre^) mice. To examine the role of ZEB1, we crossed both CRC models with either *Zeb1^+/+^* (*Z^+/+^*, WT) mice or with *Zeb1^+/–^* mice (*Z^+/–^*) (39) to generate the 4 experimental models, namely *Kras*^LSL-G12D^; *Vil1*^Cre^; *Zeb1^+/+^* (referred to hereafter as KVZ^+/+^), *Kras*^LSL-G12D^; *Vil1*^Cre^; *Zeb1*^+/–^ (KVZ^+/–^), *Braf*^LSL-V600E^; *Vil1*^Cre^; *Zeb1*^+/+^ (BVZ^+/+^), and *Bra*f^LSL-V600E^; *Vil1*^Cre^; *Zeb1*^+/–^ (BVZ^+/–^). As expected, *Zeb1* downregulation enhanced the survival of *Kras*-mutant mice (KVZ^+/+^ versus KVZ^+/–^) but, surprisingly, reduced the survival of mice with *Braf* mutations (BVZ^+/+^ versus BVZ^+/–^) (Figure 1A). These results suggest that *ZEB1* downregulation in the *Braf*-mutant CRC model enhances tumorigenesis.

### ZEB1 induces larger and more tumors in Kras^G12D^ mice but smaller and fewer tumors in Braf^V600E^ mice.

The downregulation of *Zeb1* in *Kras*-mutant mice (KVZ^+/–^) reduced both the number and size of the intestinal tumors formed relative to *Kras*-mutant mice with basal levels of *Zeb1* (KVZ^+/+^) (Figure 1, B and C). Surprisingly, compared with *Braf*-mutant mice with basal levels of *Zeb1*, the downregulation of *Zeb1* downregulation in that model (BVZ^+/–^) increased the total number and the size of large intestinal lesions or tumors, suggesting that ZEB1, besides having a role in tumor initiation, also contributes to tumor growth (Figure 1, B and C). Intestinal tumorigenesis in both *Kras-* and *Braf*-mutant mice was accompanied by a reduction in total BW, which remained unaffected by Zeb1 levels (Figure 1D). Taken together, these results suggest that ZEB1 has a tumor-promoting effect in *Kras*-mutant models of intestinal tumorigenesis but, contrary to expectations, a tumor suppressive effect in *Braf*-mutant counterparts.

### ZEB1 inhibits metastatic dissemination of intestinal tumors in Braf^V600E^ mice.

ZEB1 has a pro-invasive and prometastatic role in carcinomas (27–29, 34, 40). In line with the literature, KVZ^+/+^ mice did not exhibit distant metastasis (41, 42), nor did KVZ^+/–^ mice (Figure 1E). In contrast, BVZ^+/–^ mice displayed heavier livers with larger metastatic tumors than their BVZ^+/+^ counterparts (Figure 1F). Around 30% of BVZ^+/+^ mice also developed lung metastasis and BVZ^+/–^ mice exhibited more lung metastatic foci (Figure 1G). Liver and lung metastatic tumors in BVZ^+/–^ and BVZ^+/+^ mice were positive for CK20 (Figure 1, F and G), supporting their intestinal origin (43). Thus, and in contrast to the well-established role of ZEB1 as a pro-invasive and prometastatic factor, ZEB1 inhibited the metastatic liver and lung dissemination of intestinal tumors in *Bra*f^V600E^ mice.

### ZEB1 paradoxically inhibits EMT and promotes histologically differentiated tumors in Braf^V600E^ mice.

**Figure 2:**
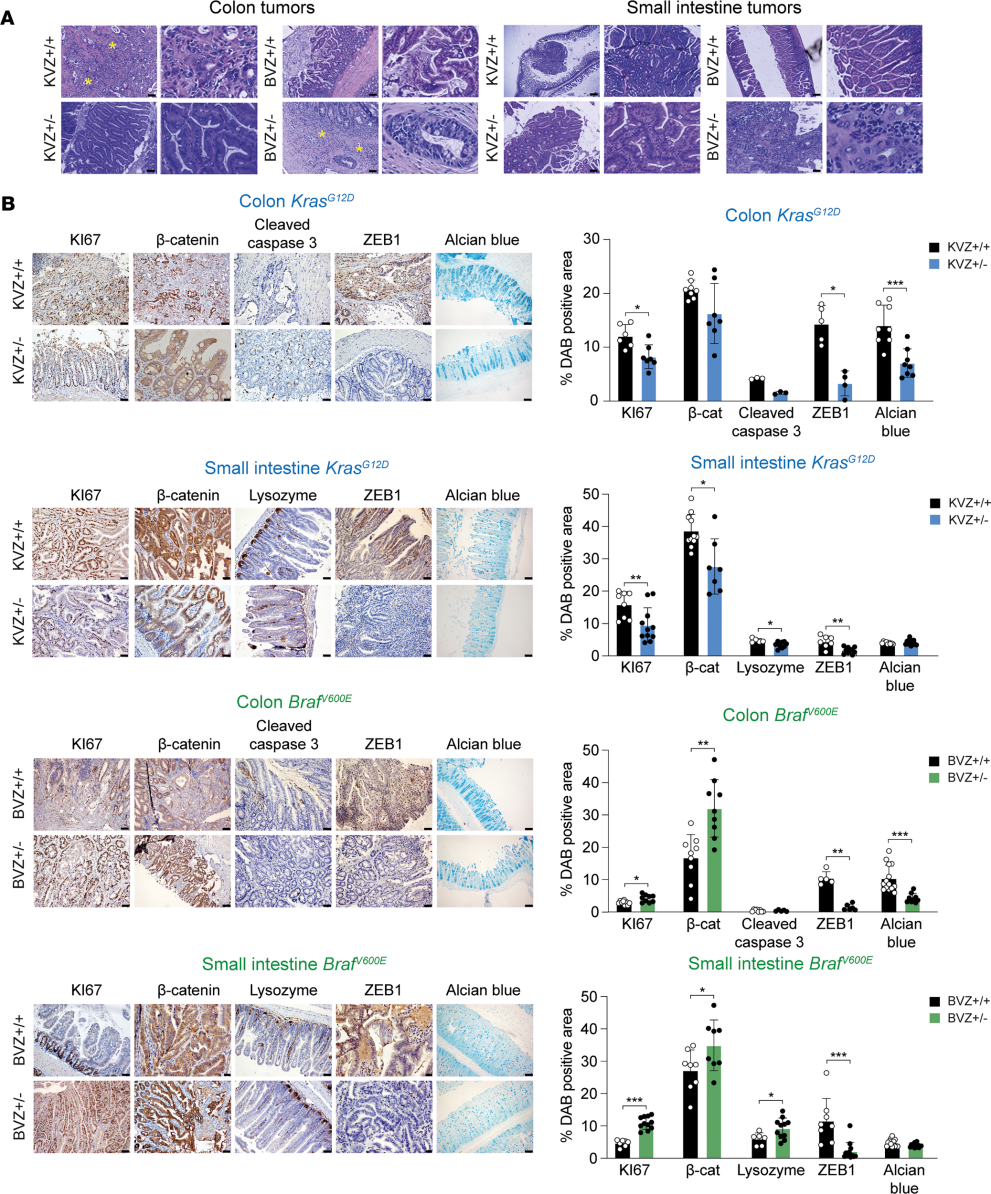
ZEB1 promotes a more differentiated histological pattern in *Bra*f^V600E^ primary intestinal tumors, whereas it induces histological dedifferentiation in *Kras*^G12D^ counterparts. (**A**) Staining for H&E and ×4 original magnifications of lesions in the colon (left panel) and small intestine (right panel) of mice of the 4 genotypes. Scale bar: 200 μm. Areas of tumor budding are marked with asterisks. (**B**) Expression of ZEB1 along with that of β-catenin (β-cat) and selected markers of proliferation (KI67), apoptosis (cleaved caspase 3), and differentiation (lysozyme, Alcian blue) in the colon and small intestine of >8-month-old KVZ^+/+^, KVZ^+/–^, BVZ^+/+^, and BVZ^+/–^ mice. Scale bars: 50 μm or 100 μm. Bar graphs are the quantification of the positive area for each marker. Respective sample numbers are as follows: for colon: KVZ^+/+^ and KVZ^+/–^ KI67 (*n* = 6, 7), β-catenin (*n* = 8, 7), cleaved caspase 3 (*n* = 3, 3), ZEB1 (*n* = 5, 4), and Alcian blue (*n* = 8, 8); for small intestine: KVZ^+/+^ and KVZ^+/–^ KI67 (*n* = 8, 11), β-catenin (*n* = 11, 7), lysozyme (*n* = 5, 9), ZEB1 (*n* = 7, 8), Alcian blue (*n* = 8, 8). Sample size numbers for colon BVZ^+/+^ and BVZ^+/–^ KI67 were 9 and 9, respectively; β-catenin (*n* = 9, 9), cleaved caspase 3 (*n* = 8, 5), ZEB1 (*n* = 5, 6), and Alcian blue (*n* = 14, 10). For small intestine, respective sample size numbers are BVZ^+/+^ and BVZ^+/–^ KI67 (*n* = 7, 11), β-catenin (*n* = 8, 8), lysozyme (*n* = 6, 11), ZEB1 (*n* = 7, 10), and Alcian blue (*n* = 11, 9). Unpaired Mann-Whitney test was used to determine statistical significance. *P* values are reported in Supplemental Table 16. ***P ≤ 0.001, **P ≤ 0.01, or *P ≤ 0.05.

We then conducted the pathological analyses of the colon and small intestine tumors generated in the different mouse models. The analysis revealed that whereas in the KVZ^+/+^ mice the tumors were mainly grade I–III adenocarcinomas, most tumors formed in the KVZ^+/–^ mice corresponded to benign hyperplasia and tubular adenomas (Figure 2A). The lesions or tumors found in the small intestine of KVZ^+/+^ mice displayed higher malignancy grades than those in KVZ^+/–^ mice. In contrast, the tumors in the small intestine of BVZ^+/–^ mice corresponded to serrated adenomas and carcinomas with a greater loss of epithelial cell polarity than the lesions found in BVZ^+/+^ counterparts. Collectively, the downregulation of *Zeb1* in *Kras*^G12D^ mice resulted in more differentiated lesions and tumors, while the downregulation of *Zeb1* in *Bra*f^V600E^ mice yielded less differentiated lesions and tumors.

High-grade tumor budding is a morphologic proxy of EMT and an independent prognostic factor associated with higher CRC recurrence, metastasis, and cancer-related death (44, 45). We found that the tumors formed in the colon of KVZ^+/+^ mice had moderate- to high-grade tumor budding (Bd2 and Bd3), whereas those in KVZ^+/–^ mice had lower or no tumor budding (Bd1) (Figure 2A). Conversely, lesions in BVZ^+/+^ mice had low tumor budding (Bd1) compared with the intermediate tumor budding (Bd2) found in BVZ^+/–^.

Intestinal tumor initiation and progression involve the deregulation of the homeostatic mechanisms controlling, *inter alia*, cell proliferation and/or apoptosis (46). In line with the reduced tumorigenesis in KVZ^+/–^ mice, the hyperplastic mucosa in these mice expressed lower levels of the proliferation marker KI67 than in KVZ^+/+^ mice (Figure 2B). Conversely, compared with lesions in BVZ^+/+^ mice, those in BVZ^+/–^ mice expressed higher levels of KI67. Relative to tumors in KVZ^+/+^ mice, tumors in KVZ^+/–^ mice exhibited lower levels of nuclear β-catenin (a marker of aberrant Wnt signaling), Alcian blue (an acidic mucin marker of goblet cells), and lysozyme (a Paneth cell marker, which is upregulated in adenomas and carcinomas; ref. 47) (Figure 2B). Conversely, BVZ^+/–^ tumors displayed higher expression of β-catenin and lysozyme than BVZ^+/+^ tumors. As in KVZ^+/–^ mice, lesions in BVZ^+/–^ had lower expression of Alcian blue. Altogether, these results suggest that ZEB1 promotes cell viability in *Kras*^G12D^ tumors but has the opposite effect in *Bra*f^V600E^ ones.

**Figure 3:**
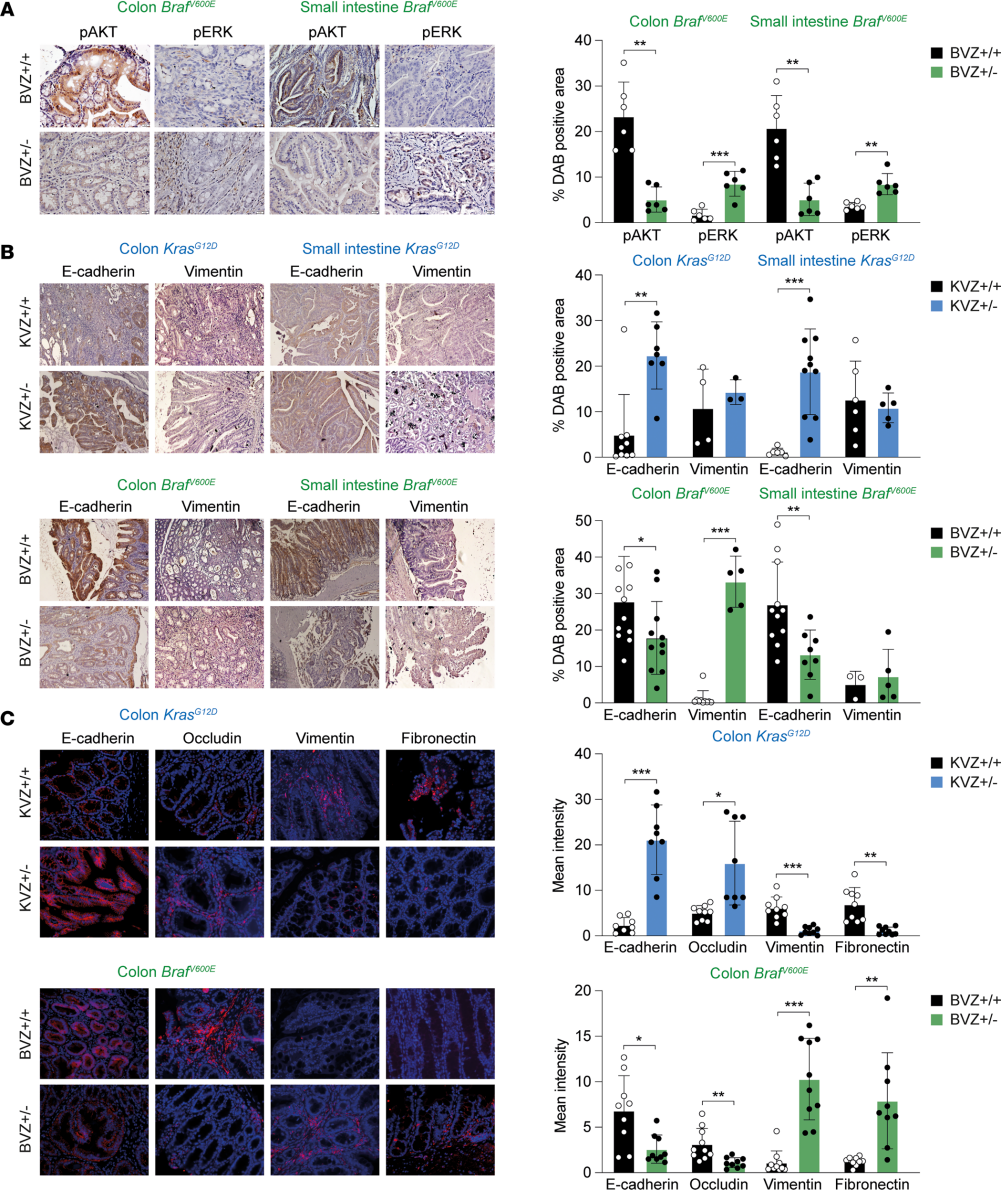
Opposite regulation of pAKT/pERK and epithelial and mesenchymal markers by ZEB1 in *Braf*^V600E^ and *Kras*^G12D^ primary CRC. (**A**) Expression of pAKT and pERK-1/2 in the colonic and small intestine (SI) lesions of >8 month-old BVZ^+/+^ and BVZ^+/–^ mice (*n* = 6), Scale bar: 20 μm. (**A**–**C**) Bar graphs are the quantification of the positive area for each marker. (**B**) IHC of E-cadherin and vimentin in the colonic and SI lesions of KVZ^+/+^ mice versus KVZ^+/–^ mice and BVZ^+/+^ mice versus BVZ^+/–^ mice. Scale bar: 100 μm. Respective sample sizes are, in colon *Kras^G12D^, n* = 9, 7 for E-cadherin and *n* = 4, 3 for vimentin, in SI, *n* = 6, 10, 6, 5; in colon *Braf^V600E^: n* = 12, 11, 10, 5 and in SI *n* = 11, 8, 3, 5. (**C**) ZEB1 regulation of epithelial and mesenchymal markers in the colonic lesions of mouse *Kras^G12D^* and *Braf^V600E^* CRC models. Immunofluorescence of E-cadherin (*n* = 8 in *Kras^G12D^;* 9 in *Braf^V600E^*), occludin (*n* = 9 and 8 in *Kras^G12D^;* 10 and 9 in *Braf^V600E^*), vimentin (*n* = 9 in *Kras^G12D^;* 10 in *Braf^V600E^*), and fibronectin (*n* = 9) (in red) counterstained with DAPI (blue) in the colon of KVZ^+/–^ (blue) and BVZ^+/–^ (green) in comparison with their WT ZEB1 counterparts (in black). Individual stainings are shown in Supplemental Figure 1A. Scale bar: 20 μm. An Unpaired *t* test was used to determine statistical significance. *P* values are reported in Supplemental Table 16. ***P ≤ 0.001, **P ≤ 0.01, or *P ≤ 0.05.

Tumors in BVZ^+/–^ mice expressed lower levels of phosphorylated AKT (pAKT) and higher of phosphorylated ERK1/2 (pERK1/2) than those in BVZ^+/+^ mice (Figure 3A). In contrast, KVZ^+/–^ tumors displayed higher expression of pERK1/2. As a driver of EMT, ZEB1 downregulates canonical epithelial genes (e.g., E-cadherin, occludin) and upregulates mesenchymal markers (e.g., vimentin, fibronectin) in different types of carcinomas (20, 48, 49). In that line, tumors and lesions of KVZ^+/–^ intestine expressed higher levels of E-cadherin and occludin and lower levels of vimentin and fibronectin than those in KVZ^+/+^ (Figure 3, B and C). Conversely, lesions in BVZ^+/–^ mice expressed lower levels of E-cadherin and occludin and higher of vimentin and fibronectin than those in BVZ^+/+^ mice (Figure 3, B and C, and Supplemental Figure 1A; supplemental material available online with this article; https://doi.org/10.1172/jci.insight.164629DS1).

**Figure 4:**
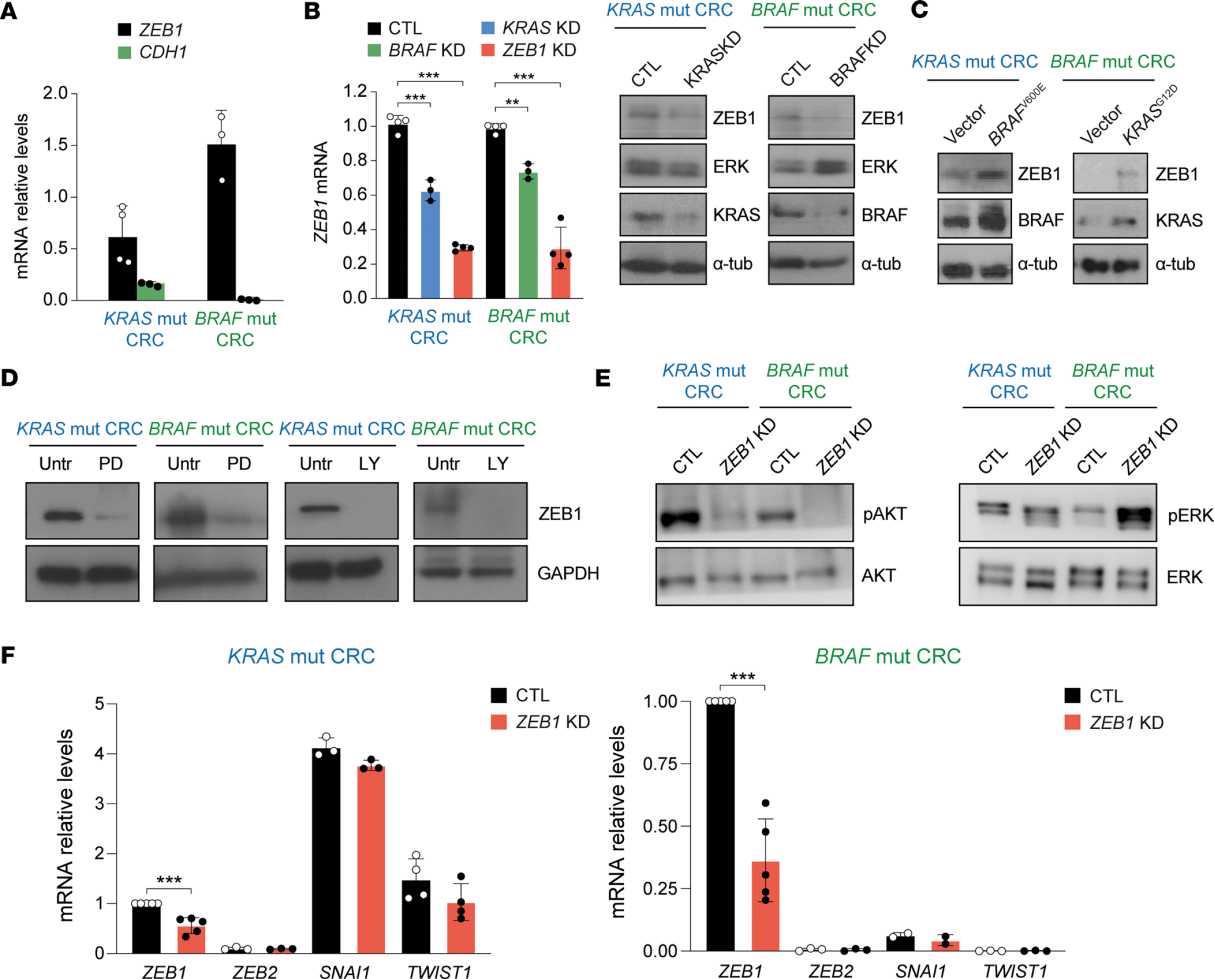
*KRAS* and *BRAF* induce ZEB1 through ERK- and AKT-dependent mechanisms. (**A**) *ZEB1* and *CDH1* mRNA in *KRAS* (LS174T) and *BRAF* (RKO)-mutant CRC cells were quantified by qRT-PCR using GAPDH as a reference gene. Bars represent the mean with the SD of at least 3 independent experiments. (**B**) *ZEB1* mRNA expression in mutant (mut) *KRAS* and *BRAF* CRC cells interfered with a nontargeting siRNA (CTL) or with specific siRNAs against *KRAS* (*KRAS* KD), *BRAF* (*BRAF* KD), or *ZEB1* (*ZEB1* KD). Dunnett’s comparison test was used. ZEB1, ERK, KRAS, and BRAF proteins were analyzed by Western blot. (**C**) ZEB1, KRAS, and BRAF proteins were analyzed by Western blot in cell lysates of *KRAS-* and *BRAF*-mutant CRC cells overexpressed with lentiviral vectors for KRAS^G12D^ and BRAF^V600E^ or an empty control vector (Vect). Α-Tubulin (α-tub) was included as a loading control. (**D**) Lysates from *KRAS-* and *BRAF*-mutant CRC cells incubated with PD98059 (PD), LY294002 (LY), or with their solvent (Untr) were immunoblotted for ZEB1 along with GAPDH as control of equal loading. (**E**) *Left:* Expression of pAKT and total AKT in *KRAS*-mutant and *BRAF*-mutant CRC cells interfered with either siCtl (CLT) or with a siRNA against *ZEB1* (*ZEB1* KD). *Right*: As in the left panel but for pERK and total ERK. (**F**) As in **A**, but for relative expression levels of *ZEB1*, *ZEB2*, *SNAI1,* and *TWIST* mRNA expression in *KRAS*-mutant and *BRAF*-mutant CRC cells stably interfered with lentivirus against *ZEB1* KD (red bar) and compared with cells interfered with by a control vector (CTL) (black bar). Expression of EMT factors was expressed relative to *ZEB1*, which was set at 100. At least 3 independent experiments were done, or ≥ 3 values were used for an unpaired *t* test of statistical significance. *P* values are reported in Supplemental Table 16. ***P ≤ 0.001, **P ≤ 0.01.

To further investigate a possible differential regulation of signal transduction by ZEB1 in *KRAS-* and *BRAF*-mutant human CRC cells, we used the LS174T (*KRAS*^G12D^, WT *BRAF*) and RKO (WT *KRAS*, *BRAF*^V600E^) CRC cell lines. RKO cells expressed higher levels of *ZEB1* mRNA than did LS174T cells (Figure 4A) and the knockdown of *KRAS* in LS174T cells or of *BRAF* in RKO cells downregulated ZEB1 expression (Figure 4B). Conversely, the overexpression of *BRAF*^V600E^ in LS174T cells or of *KRAS*^G12D^ in RKO cells upregulated ZEB1 (Figure 4C). Inhibition of either MEK signaling with the inhibitor PD98059 or of PI3K signaling with LY294002 downregulated ZEB1 protein in both cell lines (Figure 4D and Supplemental Figure 1B), suggesting that *KRAS* and *BRAF* induce ZEB1 through the same upstream MAPK and PI3K signaling pathways. Although *ZEB1* knockdown inhibited pAKT in both LS174T and RKO cells, it reduced pERK1/2 in the former but upregulated it in the latter (Figure 4E and Supplemental Figure 1C). Taken together, these data suggest that ZEB1 activates ERK signaling in mutant *KRAS* CRC cells but inhibits it when *BRAF* is mutated.

EMT factors cross-regulate each other and ZEB1 is downstream of other EMT factors (50, 51). We examined the expression of other EMT factors and whether they were differentially modulated by ZEB1 in LS174T and RKO cells. The EMT factors SNAI1 and TWIST, but not ZEB2, were expressed in LS174T cells, but all of them were barely detectable (particularly ZEB2 and TWIST) in RKO cells (Figure 4F). The downregulation of *ZEB1* did not alter *SNAI1* and *TWIST* mRNA levels in LS174T and RKO cells (Figure 4F).

### ZEB1 inhibits cell death and promotes clonogenicity and migration in KRAS^G12D^ CRC cells but not in BRAF^V600E^ CRC cells.

**Figure 5:**
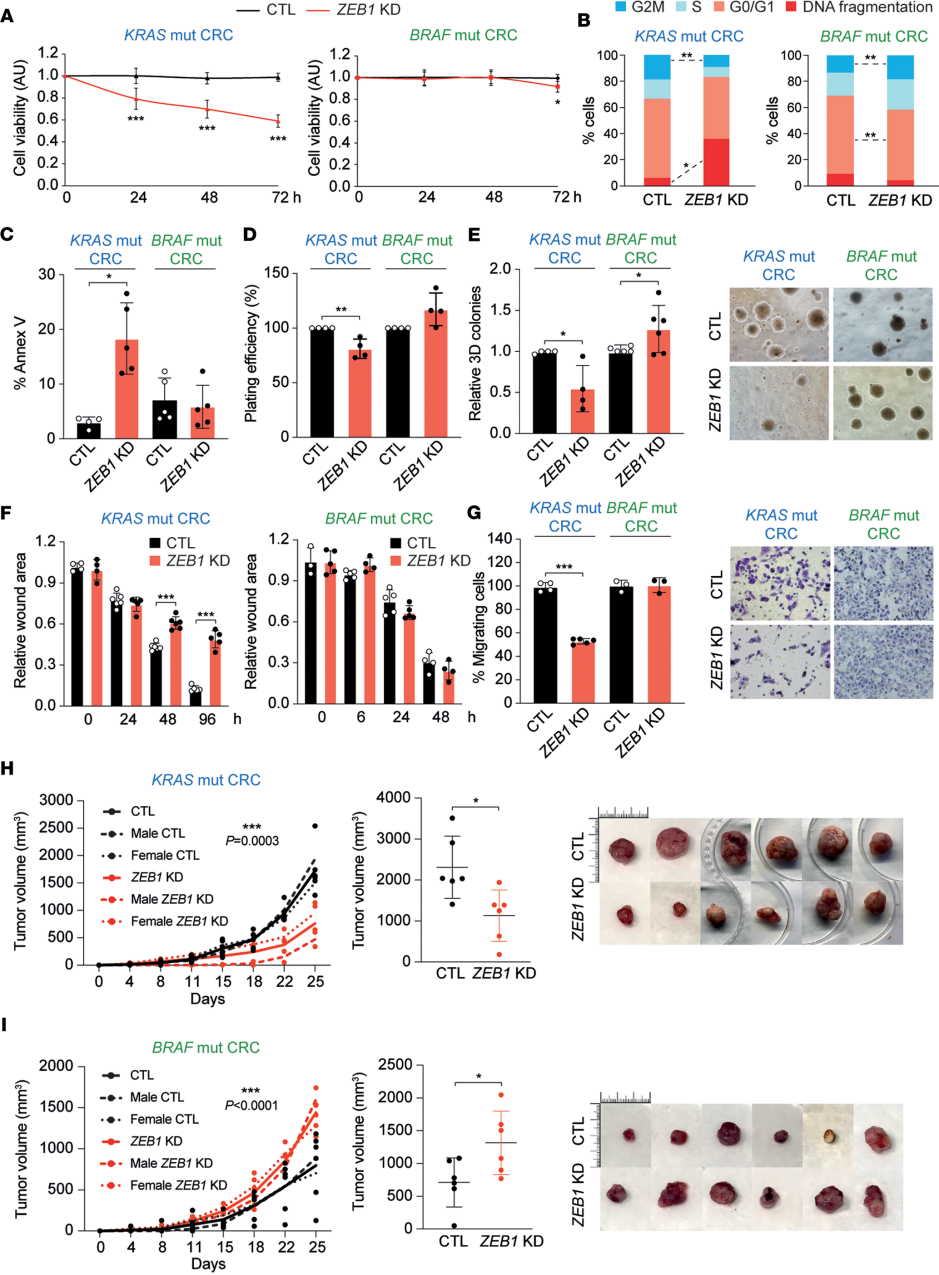
ZEB1 inhibits cell death and promotes clonogenicity, migration, and tumorigenesis in *KRAS*^G12D^ but not in *BRAF*^V600E^ CRC cells. (**A**) Cell viability of *KRAS* (LS174T) and mutant (mut) *BRAF* (RKO) CRC cells stably infected with lentivirus with an shRNA control (CTL) (black) or against *ZEB1 (*ZEB1 KD*)* (red). Cell viability by an MTT assay (*n* ≥ 5) is represented as the mean with the SD. (**B**) Opposite regulation of cell cycle progression by stable knockdown of ZEB1 in *KRAS-* and *BRAF*-mutant CRC cells. Share of cells in G2/M, S, GO/G1, or DNA fragmentation. (**C**) *KRAS-* and *BRAF*-mutant CRC cells transiently transfected with siCtl (CTL) or siZEB1 (ZEB1 KD) were assessed for apoptosis (*n* ≥ 4). (**D**) As in **C**, but for 2D clonogenicity. The plating efficiency of siCtl cells was set to 100 (*n* = 4). (**E**) 3D anchorage-independent growth of *KRAS-* and *BRAF*-mutant CRC cells as in **C**. Mean relative number of colonies with the SD (*n* ≥ 4). *Right:* Original magnification, ×100. (**F**) Cell migration in *KRAS-* and *BRAF*-mutant CRC cells, as in **C**, assessed by wound healing assays (*n* ≥ 3 in triplicate). (**G**) Cell migration in *KRAS-* and *BRAF*-mutant CRC cells, as in **F**, assessed by Transwell assays (left; *n* ≥ 3 in triplicate). (**H**) ZEB1 promotes tumorigenesis in *KRAS*-mutant CRC xenografts. *Left*: Tumor volume of 6 mice (3 males and 3 females) s.c. engrafted with cells stably infected with lentivirus encoding an shRNA control (CTL) (black) or against *ZEB1* (ZEB1 KD) (red). Two-way ANOVA test was used for comparison. *Right*: Ex vivo tumor volume and images. (**I**) ZEB1 reduces tumorigenesis in *BRAF* mut CRC xenografts. As in **H**, but with *BRAF*-mutant CRC cells. Unless stated, an unpaired *t* test was used; Bonferroni’s test was used in **A** and **F**. *P* values are reported in Supplemental Table 16. ***P ≤ 0.001, **P ≤ 0.01, or *P ≤ 0.05.

*ZEB1* knockdown reduced cell viability and cell cycle progression in LS174T cells but not in RKO cells (Figure 5, A and B, and Supplemental Figure 1D). Further analyses showed that the knockdown of ZEB1 increased apoptosis in LS174T cells but not in RKO cells (Figure 5C). ZEB1 mediates RAS/AKT-induced resistance to anoikis (anchorage-independent survival) that allows migratory cancer stem cells to shed from the primary tumor, invade the surrounding stroma, and eventually metastasize (52, 53). We found that the downregulation of *ZEB1*: (a) reduced the anchorage-dependent 2D clonogenicity of LS174T cells, whereas it slightly increased it in RKO cells (Figure 5D); and (b) inhibited the 3D cell growth of LS174T cells but promoted it in RKO cells (Figure 5E). In sum, these results suggest that ZEB1 has opposing functions on anchorage-independent cancer cell growth, promoting it in *KRAS*^G12D^ cells but inhibiting it in *BRAF*^V600E^ ones.

ZEB1 triggers a more motile phenotype in cancer cells, thus increasing their migratory capacity (27–29, 40). Accordingly, transient and stable knockdown of *ZEB1* inhibited the migration of LS174T cells in both wound healing and Transwell assays; however, ZEB1 knockdown had no significant effect in RKO cells (Figure 5, F and G).

We also tested the role of ZEB1 in the in vivo tumorigenic capacity of *KRAS-* and *BRAF*-mutant CRC cells using a xenograft model. LS174T and RKO cells with basal and downregulated levels of ZEB1 were xenotransplanted in immunodeficient nude mice and tumor formation was evaluated over time. In line with other experiments in this study, the downregulation of *ZEB1* in LS174T cells inhibited their tumorigenic capacity (tumor volume) (Figure 5H), whereas *ZEB1* downregulation in RKO cells promoted it (Figure 5I).

### ZEB1 determines different gene signatures in KRAS^G12D^ and BRAF^V600E^ CRC cells.

**Figure 6:**
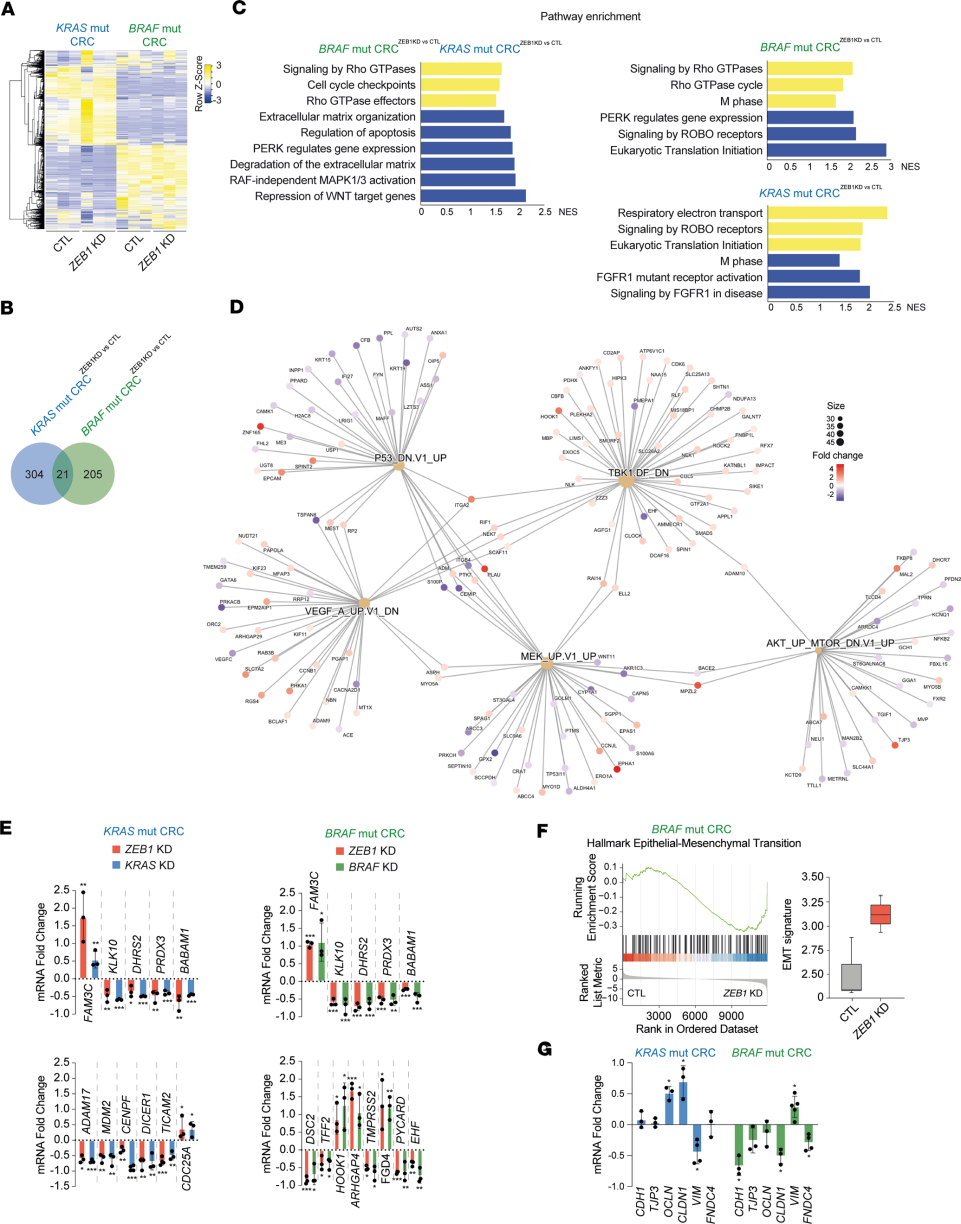
ZEB1 is associated with different gene signatures in *KRAS-* and *BRAF*-mutant CRC cells. (**A**) Heatmap of the 2499 differential expressed genes in 3 samples of LS174T^CTL^, LS174T^ZEB1KD^, RKO^CTL^, and RKO^ZEB1KD^. (**B**) Venn diagram of the DEG in LS174T^ZEB1KD^ versus LS174T^CTL^ (mutant [mut] *KRAS* CRC) and RKO^ZEB1KD^ versus RKO^CTL^ cells (*BRAF*-mut CRC). (**C**) Bar plot of normalized enrichment score (NES) of gene ontology annotations for selected signaling pathways enriched (yellow) or downregulated (blue) in RKO^CTLvsZEB1KD^ versus LS174T^CTLvsZEB1KD^ or in individual RKO^CTLvs*ZEB1*KD^ (*BRAF*-mut CRC) and LS174T^CTLvsZEB1KD^ (*KRAS*-mut CRC). (**D**) Cnet plot of RKO^CTLvsZEB1KD^ showing DEG associations. Genes are colored on the basis of the fold change associated. FDR < 0.05. (**E**) Fold-change expression of the indicated genes in *KRAS*-mutant (LS174T) and *BRAF*-mutant (RKO) CRC cells interfered with si*ZEB1* (*ZEB1* KD) (red), si*KRAS* (*KRAS* KD) (blue), or si*BRAF* (*BRAF* KD) (green) in comparison with siCtl (0 baseline). (**F**) GSEA and box plot of an EMT signature in *BRAF*-mutant CRC cells interfered with si*ZEB1* (*ZEB1* KD) in comparison with siCtl (*CTL*). (**G**) E-cadherin (*CDH1*), tight junction protein ZO-3 (TJP3), occludin (OCLN), claudin 1 (CLDN1), vimentin (*VIM*), and fibronectin III domain-containing protein 4 (FNDC4) relative mRNA expression in *KRAS*-mutant (LS174T) (blue) and BRAF-mutant (RKO) (green) CRC cells lentivirally interfered with siZEB1 (*ZEB1* KD) in comparison with CTL quantified by qRT-PCR using GAPDH as the reference gene. The 0 value line represents the value of each gene in CTL. (**E**–**G**) Bars represent the mean of ≥3 independent experiments performed in triplicate with the SD. An unpaired *t* test was used to determine statistical significance. *P* values are included in Supplemental Table 16. ***P ≤ 0.001, **P ≤ 0.01, or *P ≤ 0.05.

The gene signature associated with ZEB1 in *KRAS-* and *BRAF*-mutant CRC cells was explored by RNA-Seq. LS174T and RKO CRC cells were transiently transfected with a control siRNA or a specific siRNA against *ZEB1* (28) to generate LS174T^CTL^, RKO^CTL^, LS174T^ZEB1KD^ (where KD refers to knockdown), and RKO^ZEB1KD^ transgenic cell lines. RNA-Seq bioinformatics analysis revealed 304 differentially expressed genes (DEGs) between LS174T^CTL^ and LS174T^ZEB1KD^, and 205 DEGs between RKO^CTL^ and RKO^ZEB1KD^ cells (Figure 6A and Supplemental Table 1). There were 44 DEGs between RKO^ZEB1KD^ and RKO^CTL^ cells relative to the DEGs in LS174T^ZEB1KD^ versus LS174T^CTL^ cells. These DEGs are involved in the transcriptional regulation of pluripotent stem cells, RTK signaling, cell-to-cell junction organization, and cell metabolism (Supplemental Table 1). Importantly, of these 44 DEGs, only 21 overlapped between LS174T and RKO cells (Figure 6B), suggesting that most of the genes regulated by ZEB1 in CRC cells are specific to either *KRAS*^G12D^ or *BRAF*^V600E^ oncogenes. As with the stable downregulation of *ZEB1* (Figure 4F), the transient downregulation of *ZEB1* in both cell lines did not alter the expression of other EMT factors (e.g., SNAI1, SNAI3) (Supplemental Table 2).

Gene set enrichment analysis (GSEA) of gene ontology annotations indicated that, compared with RKO^ZEB1KD^ cells, LS174T^ZEB1KD^ cells expressed lower levels of cell cycle checkpoints and higher levels of genes associated with apoptosis and activation of pERK and RAF-independent MAPK1/3 signaling (Figure 6C and Supplemental Table 3). *ZEB1* knockdown also has opposing effects on ROBO signaling and translation initiation in *KRAS*^G12D^ versus *BRAF*^V600E^ CRC cells. DEGs regulated by ZEB1 in *BRAF*^V600E^ CRC cells include genes involved in cell signaling like *AKT*, *TBK*, *MTOR*, *MEK*, *TP53*, and *VEGF* (Figure 6D).

Several DEG that appeared commonly modulated by ZEB1 in *KRAS-* and *BRAF*-mutant CRC cells in the RNA-Seq were validated by quantitative real-time PCR (qRT-PCR); for instance, the downregulation of *ZEB1, KRAS,* or *BRAF* reduced mRNA levels of *KLK10, DHRS2, PRDX3,* and *BABAM1,* whereas it upregulated *FAM3C* mRNA (Figure 6E). Some genes were regulated by ZEB1 specifically in either *KRAS* or *BRAF*-mutant CRC cells; for instance, in *KRAS*-mutant CRC cells, ZEB1 downregulation increased *CDC25A* expression and reduced that of *ADAM17* (involved in catabolic or proteolytic processes), *MDM2*, *CENPF*, *DICER1,* and *TICAM2* (involved in cell division). In turn, in *BRAF*-mutant CRC cells, *ZEB1* downregulation increased *HOOK1* and *ARHGAP4* (cytoskeletal organizers) and *FGF4* (cell death) expression while it reduced *DSC2* and *TFF2* (cell adhesion), *TMPRSS2* (cytoskeletal organizer), *PYCARD* (cell death), and *EHF* expression (Figure 6E).

### ZEB1 inhibits the EMT signature in BRAF CRC cells and patients with CRC.

Notably, the downregulation of *ZEB1* increased the GSEA EMT signature in *BRAF*-mutant CRC cells (Figure 6F and Supplemental Table 3) where leading-edge genes of the EMT signature were upregulated (Supplemental Figure 1E). The opposing role of ZEB1 over EMT in *KRAS*- and *BRAF*-mutant CRC was validated through the mRNA assessment of epithelial—E-cadherin (*CDH1*), tight junction protein ZO-3 (*TJP3*), occludin (*OCLN*), claudin-1 (*CLDN1*)—and mesenchymal—vimentin (*VIM*) and fibronectin III domain-containing protein 4 (*FNDC4*)—genes in LS174T and RKO cells stably interfered with an shRNA against *ZEB1* or an shRNA control (Figure 6G; ref. 54). The downregulation of *ZEB1* in RKO upregulated mesenchymal markers like *VIM* and downregulated epithelial genes like *CDH1* and *CLDN1*, but it also downregulated the mesenchymal marker *FNDC4*. LS174T cells where *ZEB1* has been downregulated displayed a more epithelial phenotype, with increased expression of *OCLN* and *CLDN1* and downregulation of *VIM*. Again, these data support that ZEB1 exerts opposing effects on the regulation of the EMT program depending on the *KRAS* or *BRAF* mutational background.

**Figure 7:**
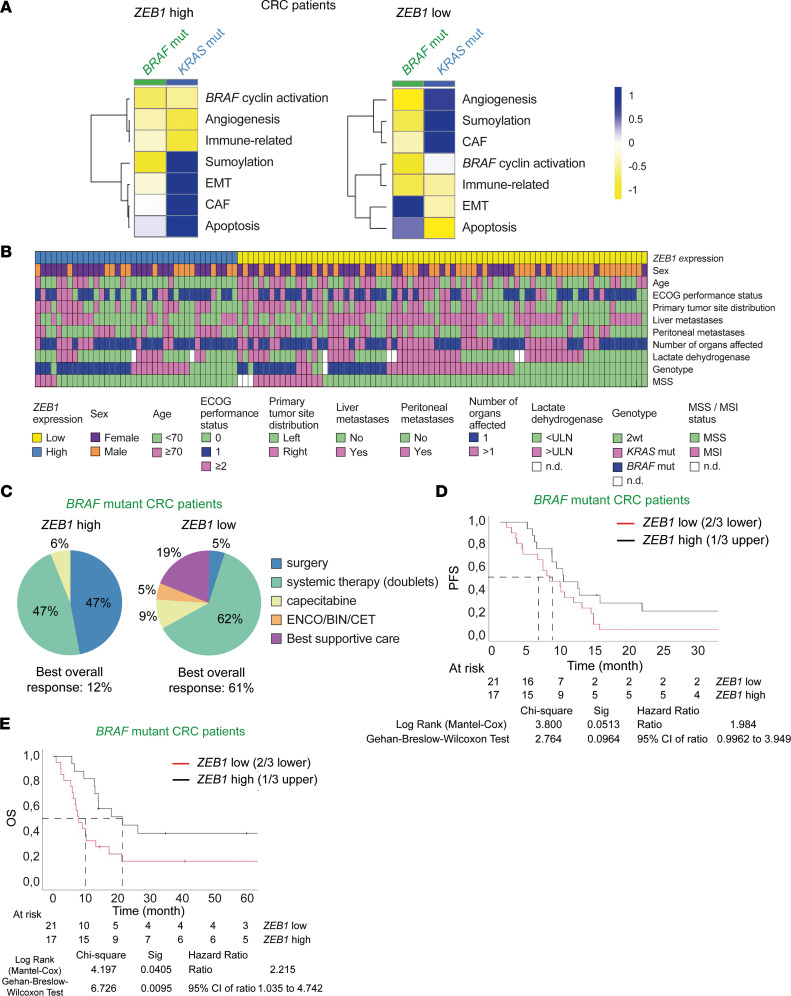
ZEB1 represses EMT and determines better survival in *BRAF*-mutant mCRC patients. (**A**) Heatmap of gene signatures in patients with *BRAF-* (green) and *RAS-* (blue) mutant (mut) mCRC with high (left) and low (right) *ZEB1* levels. (**B**) *Top panel*: *ZEB1* gene expression in the entire cohort of patients with *BRAF-* and *RAS-*mutant mCRC (*n* = 115); blue indicates high *ZEB1* expression, yellow indicates low *ZEB1* expression. *Bottom panel*: Track 1: sex (male: orange; female: purple); track 2: age (<70 years: green; ≥70 years: pink); track 3: ECOG performance status (ECOG 0: green; ECOG 1: blue; ECOG ≥2: pink); track 4: primary tumor site distribution (left side: green; right side: pink); track 5: presence of liver metastases (yes: pink, no: green); track 6: presence of peritoneal metastases (yes: pink; no: green); track 7: number of organs affected (1: blue; ≥1 organ: pink); track 8: LDH level (< upper limit of normal [ULN]: green; > ULN: pink; not determined [n.d.]: white); track 9: genotype (*KRAS*-mutant: pink; *BRAF*-mutant: blue; WT: green; n.d.: white); and track 10: microsatellite-instable/microsatellite-stable (MSI/MSS) status (MSI: pink; MSS: green; n.d.: white). (**C**) Treatment distribution and efficacy in patients with *ZEB1* high (left) and *ZEB1* low (right) *BRA*F-mutant mCRC; best overall response according to ZEB1 expression. Percentage of overall response according to each treatment is given in the figure. (**D**) Progression-free survival and (**E**) overall survival (OS) in patients with *BRAF*^V600E^ mCRC with *ZEB1* high (black line) or low (red line) according to cutoff. Univariate analysis results of overall survival in patients with *BRAF* ^V600E^ mCRC (*P* < 0.05) are reported in Supplemental Table S9, and multivariate analysis results are shown in Table 1. Sig, significance. ENCO, encorafenib; BIN, binimetinib; CET, cetuximab.

To determine whether the tumor suppressor signature and the inhibition of EMT associated with ZEB1 in *Braf*-mutant mouse models also occurred in patients with CRC, we retrospectively analyzed 41 *BRAF*-mutant mCRC according to their ZEB1 expression (Supplemental Table 4). Analyses of the gene signatures associated with human *BRAF* and *KRAS*-mutant CRC revealed that relative to *KRAS*-mutant CRC, *ZEB1* expression in *BRAF*-mutant CRC was associated with increased expression of genes related to angiogenesis, and immune and decreased apoptosis signature (Figure 7A and Supplemental Tables 5 and 6). In line with our discussed results in mouse CRC models and human CRC cell lines, we found that compared with patients with *KRAS*-mutant CRC, patients with *BRAF*-mutant CRC had a reduced EMT signature (Figure 7A).

### A high expression of ZEB1 determines better survival in patients with metastatic BRAF^V600E^ CRCs.

We then correlated the clinical characteristics and genotype distribution of 115 patients with *BRAF*-mutant mCRC with their expression of *ZEB1*. Patients whose tumors have lower *ZEB1* expression had more liver metastases, high lactate dehydrogenase levels, and a poorer overall status as determined by the Eastern Cooperative Oncology Group (ECOG) performance status scale (55). Patients with *BRAF* mutation had higher *ZEB1* expression compared with patients with *RAS*-mutant and double WT genotypes (Figure 7B and Supplemental Table 7). In line with our results in mice and human CRC cell lines, the analysis of *ZEB2*, *SNAI1*, *SNAI2*, and *TWIST1* expression in these patients did not reveal any correlation with *ZEB1* expression (Supplemental Figure 2A).

Next, to assess whether, as found in mice (Figure 1), *ZEB1* expression affects the survival of patients with *BRAF*-mutant CRC, 38 patients with metastatic CRC harboring *BRAF^V600E^* were segregated into 2 cohorts based on *ZEB1* expression above or below the upper tertile (*n* = 11 with high *ZEB1* expression [*ZEB1*-high] and 27 with low *ZEB1* expression [*ZEB1*-low]). *ZEB1*-high in patients with *BRAF^V600E^* CRC associated with metastatic resection (47% vs. 5%) and a better ECOG performance status (ECOG PS >2) (0% vs. 19%) relative to patients with *BRAF^V600E^* CRC in the *ZEB1*-low cohort (Figure 7, C and D). In fact, patients in the *ZEB1*-low cohort had a more aggressive debut of the illness (*P* = 0.004) than those in the *ZEB1*-high cohort precluding any oncologic therapy in the *ZEB1*-high group (Figure 7C and Supplemental Tables 7 and 8). The response rate was 61% in the *ZEB1*-low cohort and 12% in the *ZEB1*-high group (*P* = 0.083) (Supplemental Table 8).

In patients with metastatic *BRAF^V600E^* CRC, *ZEB1*-high was associated with better overall survival on univariate analysis and multivariate analysis (Figure 7, D and E, Supplemental Table 9, and Table 1). Interestingly, median postprogression survival in *ZEB1*-low patients was only 1 month, compared with 13 months in *ZEB1*-high patients (*P* = 0.047).

## Discussion

**Figure 8:**
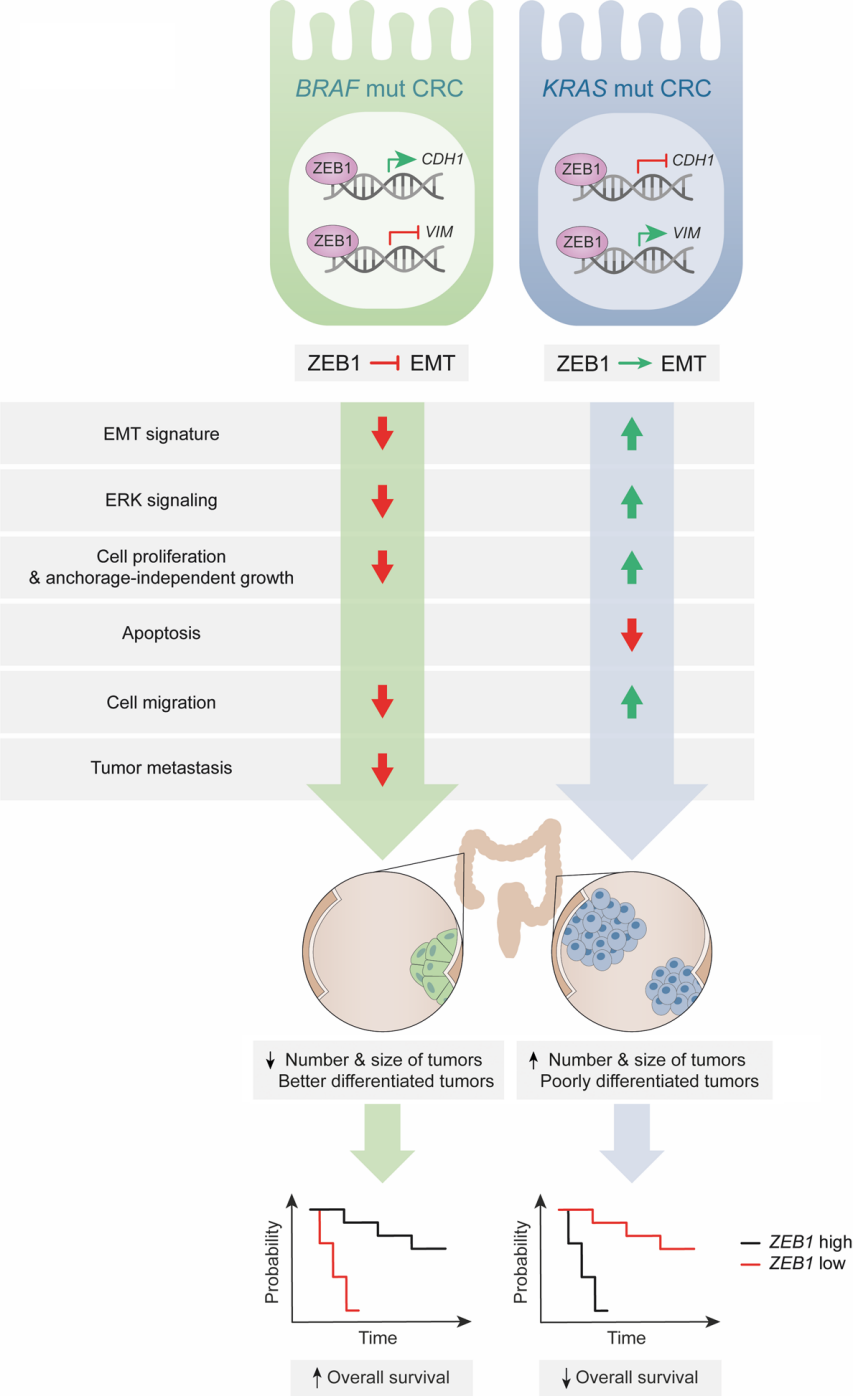
Graphical summary. ZEB1 paradoxically inhibits EMT in mutant (mut) *BRAF* carcinomas, whereas it induces EMT in *KRAS*-mutant carcinomas.

ZEB1 promotes tumor initiation and progression in both carcinomas and certain nonepithelial tumors (reviewed in refs. 13, 16, and 18). Accordingly, *ZEB1*-high associates with poorer survival in patients with CRC (22, 29) although the effect of ZEB1 based on the mutational status of CRCs had not been previously considered. Here, we found that both in human samples and mouse models of CRC, ZEB1 has a tumor-promoting role and determines poorer prognosis in mutant *KRAS* CRC but, surprisingly, it functions as a tumor suppressor and determines better prognosis in *BRAF* CRC (see schematic summary in Figure 8). In the *Kras*^G12D^ CRC mouse model, ZEB1 induced more and larger intestinal lesions and tumors with a more dedifferentiated histological pattern. Conversely, in the *Braf*^V600E^ CRC mouse model, ZEB1 determined not only fewer, smaller, and more differentiated primary CRC lesions and tumors but also fewer liver and lung metastases.

ZEB1 expression is upregulated by most developmental and oncogenic signaling pathways (e.g., Wnt, TGF-β, KRAS, Hippo, Notch); in turn, ZEB1 mediates some of the downstream protumoral functions of these pathways (27, 28, 32, 54, 56–59) (reviewed in refs. 14, 15, and 18). Our results indicate that ZEB1 is downstream of RAS/BRAF signaling and regulates ERK and AKT phosphorylation. It is worth noting that regulation of ERK is cell type specific (60); for instance, *KRAS*^G12D^ can induce the phosphorylation of ERK in Paneth cells but not in enterocytes; in turn, *BRAF*^V600E^, but not *KRAS*^G12D^, induces ERK phosphorylation in intestinal organoids. Conversely, ERK signaling also regulates ZEB1 expression (61, 62). ZEB1 mediates some of the downstream effects of RAS in cancer cells like the maintenance of a stem-like phenotype, cell proliferation, and anoikis resistance (33, 52, 63). Our results here show that ZEB1 promotes cell viability, colony formation, anchorage-independent growth, and migration in *KRAS*^G12D^ CRC cells but not in *BRAF^V600E^* CRC cells.

Although ZEB1 is best known for triggering an EMT, we found that *ZEB1*-high in *BRAF/Braf*-mutant CRCs paradoxically correlated with low tumor budding and a reduced EMT signature, the opposite than in *KRAS*-mutant CRCs. Our results also indicate that the reverse effects of ZEB1 in *KRAS*^G12D^ CRC cells but not in *BRAF^V600E^* CRC cells are not related to a differential regulation of other transcription factors known to induce an EMT (e.g., *ZEB2*, *SNAI1*, *TWIST1*).

The *BRAF^V600E^* mutation confers poor prognosis in mCRC (64); consequently, tumors aligning with the consensus molecular subtype type 1 (CMS1), which is mainly enriched with both immune cells and *BRAF* mutations, had the poorest prognosis (65, 66). In a set of patients with metastatic *BRAF*-mutant CRC treated with targeted therapy (i.e., dabrafenib, trametinib, and panitumumab), those with the BRAFV600E-mutant (BM) 2 subtype signature (BM2) (characterized by a low EMT and high oxidative phosphorylation [OXPHOS] and G2M cell cycle signatures) had the poorest prognosis (67, 68). Altogether, these clinical data suggest that, in patients with *BRAF*-mutant mCRC, the low EMT signature in the BM2 subtype could account for the poorer survival of the CMS1 subtype. A summary of published articles about BM subtype, best-observed response and survival of treated patients with *BRAF*-mutant mCRC is included in Supplemental Table 15.

The CMS4 subtype is characterized by higher *ZEB1* expression in tumors with high levels of TGF-β and with a high stromal component (69). Alternatively, in non–TGF-β–driven tumors, EMT associates with WNT signaling (70). In fact, chemotherapy resistance in CRC preclinical models relied on EMT-WNT/MYC (71, 72) and OXPHOS (73). Therefore, it can be hypothesized that in patients with *BRAF* mutation with *ZEB1*-low and whose cancer cells are exposed to a microenvironment with high competition for nutrients (e.g., CSM1; ref. 74), the increase in non–TGF-β/EMT and glycolysis/OXPHOS leads to a metabolic rewiring and poorer survival.

The expression and function of EMT factors are being targeted in several cancer therapy clinical trials, including in CRC (24). The present study highlights the need to assess the *BRAF* or *KRAS* mutational background of patients with CRC, and tentatively of other tumors, before attempting therapies targeting ZEB1. These results also stress the need to develop not only inhibitors of ZEB1 but potentially also activators of its expression and/or function.

## Methods

### Human samples.

This study includes a retrospective cohort of 115 patients with mCRC enriched with *BRAF* mutations who were diagnosed at the Hospital Clínic of Barcelona (Barcelona, Spain). Eligibility criteria and basic clinical data of patients with mCRC are detailed in the Supplemental Methods.

### Mouse models.

The following mouse models were used in the study: C57BL/6J (denoted as *Zeb1^+/+^*), *Zeb1^+/–^*, *Kras*^LSL-G12D^, *Braf*^LSL-V600E^, and *Vil1*^Cre^. The last 3 models were purchased from The Jackson Laboratory. See the Supplemental Methods for additional details on these mice and their crossing. Xenograft studies were performed using athymic nude mice purchased from Charles River Laboratories. The list of primers used for mouse genotyping is detailed in Supplemental Table 10.

### Cell lines and cell culture.

LS174T and RKO CRC cells were cultured as described in Supplemental Methods. Where indicated, cell lines were stably or transiently interfered for *ZEB1, KRAS,* or *BRAF* using shRNA harboring lentiviral vectors or siRNA oligonucleotides, respectively, as described in the Supplemental Methods. The sequences of shRNA lentiviral constructs and siRNA oligonucleotides are included in Supplemental Tables 11 and 12, respectively. LS174T and RKO cells stably interfered with either a noncoding shRNA control or an shRNA specific against *ZEB1* are referred to here as LS174T^CTL^, RKO^CTL^, LS174T^ZEB1KD^, and RKO^ZEB1KD^, respectively.

### Cell viability and clonogenic assay.

Cell viability, proliferation, apoptosis, clonogenic, and migration assays were assessed as detailed in Supplemental Methods.

### Determination of protein and RNA levels.

Determination of protein expression by Western blot and/or immunostaining is described in Supplemental Methods. The identities and sources of primary and secondary Abs are included in Supplemental Table 13. Relative mRNA levels were determined by qRT-PCR. The DNA primers used in the qRT-PCR are included in Supplemental Table 14.

### Bulk RNA-Seq and NanoString gene expression profiling.

Gene expression profiles were assessed by RNA-Seq. A NanoString panel was used to interrogate gene expression on FFPE tissue. All procedures are detailed in Supplemental Methods.

### Data availability.

The RNA-Seq data have been deposited in the NCBI Gene Expression Omnibus under reference GSE123416.

### Statistics.

Statistical analysis was performed using SPSS 18.0 (IBM), SPSS 17.0 (IBM), or GraphPad Prism 8.0.1 (GraphPad Software). The type of statistical test used and the corresponding *P* value is indicated in Supplemental Table 16. Unless specified otherwise, the means and SD of data and the statistical significance of their differences were assessed with a nonparametric, unpaired Mann-Whitney test and 2-tailed Student’s *t* test. Statistical analyses involving multiple comparisons relative to a shared control were carried out with a 95% CI using Dunnett’s test or Tukey’s test (when also including comparison between noncontrol conditions). Bonferroni’s test with a 95% CI was used for time-specific comparisons. Xenograft volume analysis was analyzed with a 2-way ANOVA test. Qualitative variables such as demographic and clinical variables were analyzed with a χ^2^ test to compare the groups of patients with high and low expression of ZEB1. In Kaplan-Meier survival analyses, differences in mouse (Figure 1, A and B) and patient (Figure 7, D and E) survival probabilities were determined by log-rank test and Mantel-Cox methods using SPSS (IBM) and SAS software. Logistic regression analysis was used to identify possible explanatory variables involved in survival. In the analysis of progression-free survival, data from patients who were alive without disease progression were censored as of the time of the last imaging assessment. Radiological progression and death that occurred without disease progression were included as events. Postprogression survival was calculated from the time of progressive disease to the date of death or last follow-up. For the analysis of overall survival, data for patients without documented death at the date of cutoff were censored. In turn, censored mice refers to those euthanized at the indicated periods to harvest and analyze their tissues. Where appropriate, relevant comparisons were labeled in figures as significant at the following values: ***P ≤ 0.001, **P ≤ 0.01**, or *P ≤ 0.05. *P* values were nonsignificant when *P* > 0.05. The *P* values reported in all figures also are given in Supplemental Table 16.

### Study approval.

The use of human samples in the study was approved by the Clinical Ethics Research Committee at the Hospital Clinic of Barcelona (references HCB-2013/8674, HCB-2018/0633, and HCB-2019/0255). All patients and donors gave their informed consent for the use of samples in accordance with the principles of the Helsinki Declaration. The use of mice in the study followed the guidelines of the Animal Experimental Committee at the University of Barcelona School of Medicine and was approved under references CEEA 347/14 and 193/16.

## Author contributions

EST performed most of the experimental work described in the article and designed specific experiments. LP and BG conducted bioinformatics analysis of survival data. IV provided technical support for the overall experimental work and in the maintenance of the mouse colony. YC carried out some qRT-PCR and IHC analyses. LSM assisted with some in vivo experiments. AEC, JJL, and LS carried out the RNA-Seq bioinformatics analysis. MC supervised pathological examinations. DSD, JM, and AC provided critical reagents for the study. AP conceived, designed, and supervised the study. EST and AP wrote the manuscript, which was critically reviewed by all authors.

## Supplementary Material

Supplemental data

Supporting data values

## Figures and Tables

**Table 1:**
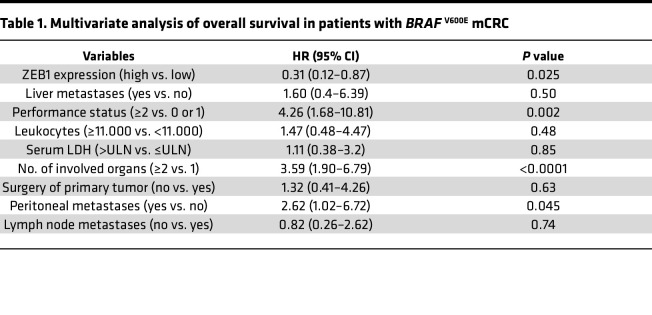
Multivariate analysis of overall survival in patients with *BRAF* ^V600E^ mCRC

## Version 1 10/23/2023

Electronic publication
