# Supplementary material for: The EMT factor ZEB1 paradoxically inhibits EMT in BRAF-mutant carcinomas
Source: JCI Insight. 2023 Oct 23;8(20):e164629. doi: 10.1172/jci.insight.164629 (PMC10619495; doi:10.1172/jci.insight.164629)

SUPPLEMENTARY INFORMATION

FOR

The EMT factor ZEB1 paradoxically inhibits EMT in *BRAF* mutant carcinomas

Ester Sánchez-Tilló[1,2,3,*], Leire Pedrosa[4#], Ingrid Vila[1#], Yongxu Chen[1#], Balázs Győrffy[5#], Lidia Sánchez-Moral[1], Laura Siles[1], Juan José Lozano[6], Anna Esteve-Codina[7], Douglas S. Darling[8], Míriam Cuatrecasas[3,9], Antoni Castells[2,3,10], Joan Maurel[3,4], Antonio Postigo[1,3,11,12,*]

[1] Group of Gene Regulation in Stem Cells, Cell Plasticity and Differentiation in Cancer, Dept. of Oncology and Hematology, IDIBAPS, Barcelona 08036, Spain.
[2] Group of Gastrointestinal and Pancreatic Oncology, Dept. of Liver, Digestive System and Metabolism, IDIBAPS, Barcelona 08036, Spain.
[3] Biomedical Research Network in Gastrointestinal and Liver Diseases (CIBEREHD), Carlos III National Health Institute (ISCIII), Barcelona 08036, Spain.
[4] Group of Translational Genomics and Targeted Therapeutics in Solid Tumors, IDIBAPS, and Dept. of Medical Oncology, Hospital Clinic, Barcelona 08036, Spain.
[5] TTK Cancer Biomarker Research Group, and Dept. of Bioinformatics and 2nd Dept. of Pediatrics, Semmelweis University, Budapest, Hungary.
[6] Bioinformatics Platform. Biomedical Research Network in Gastrointestinal and Liver Diseases (CIBEREHD), Carlos III National Health Institute (ISCIII), Barcelona 08036, Spain.
[7] CNAG-CRG, Center for Genomic Regulation (CRG), The Barcelona Institute of Science & Technology (BIST), Barcelona 08028, Spain, and Universitat Pompeu Fabra (UPF), Barcelona 08002, Spain.
[8] Dept. of Oral Immunology, and Center for Genetics and Molecular Medicine, University of Louisville, Louisville, KY 40202, USA.
[9] Group of Molecular Pathology of inflammatory conditions and solid tumours, Dept. of Oncology and Hematology, IDIBAPS, Barcelona, 08036, Spain and Dept. of Pathology, Hospital Clínic and University of Barcelona School of Medicine, Barcelona 08036, Spain.
[10] Dept. of Gastroenterology. Hospital Clinic and University of Barcelona School of Medicine, Barcelona 08036, Spain.
[11] Molecular Targets Program, Dept. of Medicine, J.G. Brown Cancer Center, Louisville, KY 40202, USA
[12] Catalan Institution for Research and Advanced Studies (ICREA), Barcelona 08010, Spain.

# LP, IV, YC and BG contributed equally as co-second authors to the work

* **Corresponding authors**: E Sánchez-Tilló (esanche3@recerca.clinic.cat, Phone: +34932275400 ext 4560) & A Postigo (idib412@clinic.cat, Phone: +34932275400 ext 3325). IDIBAPS. Rosselló 149-153, Barcelona 08036, Spain

Running Title: Opposing roles of ZEB1 in *KRAS* and *BRAF* mutant CRCs

Keywords: Colorectal carcinoma / mutant *KRAS* / mutant *BRAF* / ZEB1 / EMT / RAS-MAPK signaling

SUPPLEMENTARY METHODS

Transgenic mouse models

The following mouse models were used in this study. B6.129P2 (Cg)-Braftml1Mcmcm/J (reference 017837) (1), B6.129S4-Krastm4Tyj/J (reference 008179) (2) and B6.Cg-Tg (*Vil1*^{Cre}) 1000Gum/J (reference 021504) (3) mice harboring a Cre-activated *Braf*^{V600E}, Cre-activated *Kras*^{G12D}, and Cre recombinase under the mouse *Vil1* promoter, respectively, were purchased from Jackson Laboratory (Bar Harbor, ME). *Vil1*^{Cre} recombination restricts expression in villi and crypts of epithelial cells of the small and large intestine. No expression of the transgenes was found in other tissues. These mice were crossed with ZEB1$^{+/-}$ mice (4) to yield *Kras*$^{LSL\text{-}G12D}$; *Vil1*^{Cre}; *Zeb1*$^{+/+}$ (KVZ+/+), *Kras*$^{LSL\text{-}G12D}$; *Vil1*^{Cre}; *Zeb1*$^{+/-}$ (KVZ+/-); *Braf*$^{LSL\text{-}V600E}$; *Vil1*^{Cre} *Zeb1*$^{+/+}$ (BVZ+/+) and *Braf*$^{LSL\text{-}V600E}$; *Vil1*^{Cre}; *Zeb1*$^{+/-}$ (BVZ+/-) and were compared to *Kras* wild-type/*Braf* wild-type; *Vil1*^{Cre}; *Zeb1*$^{+/-}$ (Z+/-) and/or completely wild-type *Kras* wild-type/*Braf* wild-type; *Vil1*^{Cre}; *Zeb1*$^{+/+}$ (Z+/+). *Zeb1*$^{+/-}$ mice were used since homozygous animals die perinatally by respiratory failure, skeletal defects and impaired thymic T cell development. Phenotypic effects of *Zeb1*$^{+/-}$ have been observed in cancer (4). Mice survival endpoint criteria include a scoring system to establish disease progression and ethical sacrifice based on weight measure and observation (body posture, appearance, activity level, prolapse and diarrhea). Before tissues were collected at the indicated times, animals were weighed and the small intestine, colon, lung and liver were collected and washed several times with PBS. Length, weight and tumor dimensions were measured

previous to o/n 10% formalin-fixing and embedding in paraffin. Evaluation of mice biopsies was carried on blind by Patconsult BCN. SL. (Barcelona, Spain) and IDIBAPS' Tumor Bank core facility. DNA for genotyping was obtained from mouse ears with 25 mmol/L NaOH/0.2 mmol/L EDTA extraction at 98ºC, 1 h plus 40 mmol/L Tris-HCl pH 5.5 as described previously (4). Primers used in PCR are described in Supplementary Table S11. PCR conditions were as follows: 10 cycles of 94ºC 30 s, 64ºC 30 s and 68ºC 10 s plus 32 cycles of 94ºC 15 s, 60ºC, 30 s and 72ºC 10 s. *δEF1*-null-lacZ monoallelic inactivated mice [$Zeb1^{+/-}$] were genotyped for WT using Pro1 and TM1 and mutant using Pro1 and LacZ-B primers. PCR conditions for *Zeb1* were as follows: 35 cycles of 94ºC for 30 s, 60ºC for 1 min and 72ºC for 40 s. PCR was performed either using AccuStart II Taq DNA polymerase (Quanta Biosciences) or Dream Taq DNA polymerase (Life Technologies, Thermo Fisher Scientific, Carlsbad, CA) or Maxime TaqDNA polymerase (Intron Biotechnology) with 2 mmol/L MgCl2, 0.2 mmol/L dNTPs and 1.25 µmol/L primers in a final volume of 25 µl. DNA was separated in 2% agarose gel with RedSafe Nucleic Acid Staining Solution (INtRon Biotechnology) in TBE (TrisBase, Boric Acid, EDTA) at 100 V for 30 min. Animal survival was plotted using Kaplan Meier survival curve as indicated in the statistic section.

Xenograft mouse models

Xenograft studies were performed using both three 4-week male and three female outbred Athymic Nude (Crl:NU(NCr)-$Foxn1^{nu}$ obtained from backcrossing of male homozygous athymic nude (strain code 490, Charles River) and female

heterozygous athymic nude (strain code 491, Charles River). A total of $2x10^6$ LS174T or RKO cells interfered with an shRNA control (CTL) or an shRNA specific for *ZEB1* (*ZEB1*KD) mixed in 1:1 dilution with 50% Cultrex reduced growth factor basement membrane extract type 2 (BME2) (R&D Systems) were subcutaneously injected in flanks of the same mice. Tumor volume calculated as length x height^2 x 0.5236 was measured every 3-4 days with a caliper. When tumors reached 1.5cm^3, mice were euthanized and tumor volumes (length x width x height) were measured *ex vivo*.

Human CRC patients and samples

This study includes a retrospective cohort of patients diagnosed with *BRAF* mutant and *KRAS* mutant metastatic CRC between February 2016 and February 2020 at the Hospital Clínic of Barcelona. Data collection of patients was censored on November 2020. Clinical-pathologic information of these patients was prospectively collected from the registry database at the Hospital Clínic of Barcelona and summarized in Supplementary Table S4. Key eligibility criteria included a diagnosis of stage IV disease with histologically confirmed adenocarcinoma within the colon or rectum. Clinical parameters utilized to initially evaluate the patient's performance were: sex, age, Eastern Cooperative Oncology Group (ECOG) performance status, stage at diagnosis and metastatic organ type.

Targeted next generation sequencing of *BRAF* and *KRAS* mutant CRCs

The Oncomine Solid Tumor DNA kit was used to sequence a panel of 22 genes, which include commonly mutated genes in CRC except for the *APC* gene. Tumor DNA was extracted from formalin-fixed paraffin-embedded (FFPE) unstained sections of primary CRC specimens, using the QIAmp® DNA FFPE kit (Qiagen, Hilden, Germany) and then quantified with Qubit® fluorometer assay (Thermo Fisher Scientific, MA, USA). Amplicon sequencing libraries were prepared from 10 ng of input DNA per sample (minimum of 0.83 ng/µl DNA concentration) using specific primers from the Oncomine Solid Tumor DNA kit (Thermo Fisher Scientific, MA, USA). Libraries were sequenced using the Ion Torrent Personal Genome Machine platform (ThermoFisher Scientific) at the Genomics Facility at IDIBAPS. Data were analyzed using the Ion Reporter Software (ThermoFisher Scientific) according to the manufacturer's instructions. Mean coverage was 500X. Raw data files processing and downstream analyses were performed using the Torrent Suite Software version 4.0.2 automated pipeline, including adapter trimming, QC and alignment to GRCh37/hg19 reference genome. Single-nucleotide variants (SNVs) and short insertions and deletions (indels) were identified and annotated with the Ion Reporter Software (ThermoFisher Scientific) according to the manufacturer's instructions All detected variants were filtered and prioritized according to a minimal coverage depth of 100X, variant allele frequency (VAF) of at least 4%, a frequency in normal population <1% based on ExAC, presence in COSMIC and functional pathogenicity consequences reported in the ClinVar database.

CRC cell lines and cell culture

Colorectal cancer (CRC) cell lines were obtained from the Cancer Cell Line Repository at the Barcelona Biomedical Research Park (Barcelona, Spain), which conducts routine tests for cell line authentication. LS174T and RKO cells were cultured in Dulbecco's modified Eagle medium (DMEM) (Lonza, Basel, Switzerland) with 10% Fetal Bovine Serum FBS (Sigma-Aldrich, St. Louis, MO) and 1% Penicillin-Streptomycin (Lonza). Cells were trypsinized with 0.05% trypsin/EDTA solution (Lonza). Cells were periodically tested for mycoplasma with e-Myco TM plus Mycoplasma PCR detection kit (iNtRON Biotechnology, Gyeonggi-do, South Korea). Wherever indicated, cells were treated for 24 h with 25 μmol/L PD98059 (Calbiochem, Millipore) or 50 μmol/L LY294002 (Tocris-Biotechne, Bristol, UK) dissolved in DMSO.

Stable and Transient Transfection and/or Interference of CRC cell lines

Plasmid DNA isolation was performed using PureYield Plasmid Maxiprep System (Promega) and integrity was checked in Nanodrop and 1% agarose gel. For lentiviral production, 16 x 10^6 293T cells in 150 cm plates were used to transfect 20 μg vector hairpin FCIV or FCIV-*BRAF*^{V600E}-His-Amp^R (gifted by Dr. J. Verdaguer, Immunology and Immunopathology Group (GRIIP), Lleida University, Lleida, Spain) or puro*KRAS*^{G12D} (gifted by S. Barbacid, CNIO, Madrid, Spain) along with 10 μg envelop plasmid pMD2G [a gift from Didier Trono, (EPFL, Lausanne, Switzerland) (Addgene plasmid # 12259; http://n2t.net/addgene:12259; RRID:Addgene_12259)] and 10 μg packaging plasmid pCMV-dR8.2 dvpr [a gift from Bob Weinberg

(Massachusetts Institute of Technology, MA, USA) (Addgene plasmid # 8455; http://n2t.net/addgene:8455; RRID:Addgene_8455)] using Lipofectamine 2000 or 3000 (Invitrogen ThermoFisher, Carlsbad, CA) and OPTIMEM. Cells were incubated o/n and viral supernatants were collected after 72 h and filtered using a 0.45 µm filter. Supernatants were centrifuged at 25,000 rpm for 90 min and the pellet was resuspended. Supernatants were used to infect indicated 50% confluent cells in 12 well plates in the presence of 5 µg/ml polybrene (Sigma-Aldrich). Stable transfection was positively selected in 10 µg/ml Puromycin (Sigma-Aldrich) every 3 d for 2 wk. Stable lentiviral infections of *ZEB1* were a pool of three shRNAs against human *ZEB1* or an shRNA control, whose sequence is described in Supplementary Table S12 in parallel to shCtl lentivirus. For siRNA transfection with Lipofectamine RNAiMAX (ThermoFisher, Waltham, MA), 70% confluent cells were transfected with 100-200 nmol/L siRNA complexes with RNAiMAX using OPTIMEM media (Gibco) in the presence of antibiotics. siRNA used are described in Supplementary Table S13. siRNAs used were transfected in parallel to a control siRNA siCtl. After 48-72 h, DNA- or siRNA/shRNA-transfected cells were collected for analysis.

Tissue Immunostaining

Slides of 4 µm thickness from mice paraffinized tissues were sectioned at the IDIBAPS' Biobank core facility using a Microtome (Leica Biosystems) with feather R35 disposable blades (Casa Alvarez, Madrid, Spain) and poly-L-lysine slides Menzel Gläser (VWR). Human and mouse biopsies were xylene deparaffinized and hydrated in sequential EtOH:H2O dilutions. After epitope heat retrieval for 5 min with

10 mmol/L Sodium citrate pH 6 and 15 min endogenous peroxidase blockage with 0.3% H2O2/MeOH (for IHC) or 0.1% sodium borohydride $NaBH_4$ (for IF), blocking was performed with 5% goat or donkey serum (Jackson ImmunoResearch Europe) in PBS/0.5% Tween/4% BSA buffer. It was followed by primary Ab incubation in blocking buffer overnight at 4ºC or 1 h at r.t. Staining was detected using corresponding donkey anti-mouse, rabbit or goat secondary IgG (H+L)-HRP (Jackson ImmunoResearch Europe) at 1:200 dilution, 1 h at 37ºC in blocking solution followed by DAB chromogen kit (Vector Labs, Burlingame, CA) or NeoLink-2 Plus HRP broad with DAB kit (CliniSciences). After counterstaining with Gill's Hematoxylin (Sigma-Aldrich), slides were dehydrated and mounted with DPX Mountant for histology (Sigma-Aldrich). For IF, goat IgG-Cy3 was used (at 1:200 dilution, 1h at 37ºC in blocking solution) followed by DAPI counterstaining and mounting with Fluorlast mounting media (Quimigen). H&E staining was performed by incubating hydrated tissues with H&E followed by EtOH/LiCl staining and washing with EtOH. For Goblet cell detection, slides were stained with Alcian blue pH 2.5 mucin stain (Abcam) for 30 min at r.t. followed by staining with O safranin solution. Photographs were collected using a bright field Olympus BX41TF microscope and Cell Sens software (Olympus America Inc., Melville, NY). The mean intensity or % of DAB area were quantified using Image J or Fiji by deconvoluting images for HE DAB, adjusting the threshold and applying a watershed in binary images. The antibodies used in the study are in Supplementary Table S14.

Western blot assays

Cells were lysed using RIPA lysis buffer (150 mol/L NaCl, 50 mmol/L Tris HCl pH 7.4; 0.5% SDS, 0.5% NaDeoxycholate, 1% NP40 and 0.8 mmol/L EDTA pH 8) with protein inhibitors (10 µg/ml Leupeptin, Aprotinin, Pepstatin and PMSF). Protein supernatants were quantified using A562 nm with Pierce BCA Protein assay kit (ThermoFisher) or A595nm with Bradford kit (Bio-Rad). Lysates were boiled, loaded into 12% acrylamide separating gel and transferred to PVDF membranes (Immobilon-P, Millipore, Bedford, MA) using CAPS buffer pH 11 + 10% MeOH. After blocking for 1 h at r.t. with 5% non-fat milk in TBST (137 mol/L NaCl, 20 mmol/L Tris, 0.1% Tween-20, pH 7.6), primary Abs in TBST were incubated at 4ºC o/n or 1 h at r.t. Detection was performed using secondary Peroxidase-AffiniPure donkey anti-mouse IgG (H+L), peroxidase-AffiniPure goat anti-rabbit IgG (H+L) (1:5000-10000) (JIR-Jackson ImmunoResearch Europe, Newmarket, UK) for 1 h at r.t.. The chemiluminescent reaction was developed using ClarityTM ECL Western Blotting Substrate (Bio-Rad, Hercules, CA) and X-Ray Films (Rosex). The antibodies used in the study are listed in Supplementary Table S11. Western figures are representative of at least three independent experiments.

RNA isolation, RT-PCR, real-time PCR

Total RNA from cell lines was obtained with RNAzol® RT reagent (Sigma-Aldrich) or Purezol® (Bio-rad) by Isopropanol precipitation and EtOH washing and was reverse transcribed. Total RNA was quantified, and its quality was checked by Nanodrop 2000c (ThermoScientific) at A260/230 >1.6 and A260/280 >1.8. After

DNAse treatment of RNA following the manufacturer's protocol (Promega), cDNA was synthesized using iScript cDNA synthesis kit (Bio-Rad) or SuperScript IV Reverse Transcriptase (Invitrogen Life Technologies) or High Capacity cDNA reverse transcription kit (Life Technologies) following manufacturer's instructions using random primers. qPCR reactions were performed either in a Light Cycler 96 (Roche) or in a Bio-Rad Chromo-4 real-time PCR machine using iTaq Universal SYBR Green supermix (Bio-Rad) or GoTaq® qPCR Master Sybr Green Mix (Promega) with the following conditions: 95ºC for 30s, 38 cycles of 95ºC for 15 s followed by 60ºC for 30 s. RT-PCR was performed using Master Sybr Green Mix. Data were analyzed with Light Cycler 96 (Roche) or Opticon Monitor v3.1.32 software (Bio-Rad) for absolute quantification by ΔΔCt method normalizing values with *GAPDH* as housekeeping gene and Melting curve analysis. Graphs of RT-PCR show the average of a minimum of three experiments run in triplicate. Oligonucleotides were previously published in indicated reference or in pga.mgh.harvard.edu/primerbank/ and PrimerDepot database. They were purchased from Sigma-Aldrich, IdT Costar or ThermoFisher Scientific and their sequences are detailed in Supplementary Table S15.

RNA sequencing and bioinformatics analysis

For RNA sequencing (RNAseq), total RNA was isolated and its quality (RIN 8-10) was checked using Agilent 2100 Bioanalyzer (Agilent, Santa Clara, CA) at Genomic Platform. Quantitative determination of differentially expressed genes by RNAseq was done with Stranded mRNA-seq >20M reads (HiSeq 2000, 2x75 bp read length)

(Illumina Inc., San Diego, CA) in triplicate at National Center of Genomic Analysis (CNAG, Scientific Park of Barcelona). RNA-seq reads were mapped against the human reference genome (GRCh38) using STAR (version 2.5.3a) (22) with ENCODE parameters. Genes were quantified using RSEM (version 1.3.0) (5) with default parameters. The human gene annotation file was downloaded from Gencode release 35 (https://www.gencodegenes.org/). Mapping and quantification quality checking was performed with GEMTools (https://gemtools.github.io/) and custom Python scripts. Differential gene expression was performed with 'limma-voom' (6) and the function 'duplicateCorrelation' for intrablock correlation. Genes with FDR<5% were considered differentially expressed between conditions. Gene set enrichment analysis was done with the fgsea R package (7) with a pre-ranked "limma t-statistic" gene list. Category Netplot (cnetplot) was done with clusterProfiler in R with DEG from RNAseq (8). A category network was performed with the oncogenic signature gene set from GSEA. Boxplot of the ssGSEA (9) score for the EMT signature were built. Welch student t-test were employed to check statistical significance. The RNAseq is available in National Center for Biotechnology Information (NCBI)'s Gene Expression Omnibus (GEO) with accession number GSE123416.

Cell viability, cell cycle and apoptosis assays

For cell viability MTT assays, $1x10^4$ cells were seeded in 96 well plates in triplicate per condition and incubated during 4 d. 5 mg/ml MTT (Sigma-Aldrich) was added and incubated for 1-4 h at 37ºC till the formation of formazan blue precipitates. After

adding DMSO, A560 nm for MTT and A750 nm for background noise were measured using the Modulus II Glomax microplate reader (Promega). The absorbance of samples minus blank Abs was represented versus the dose or day of treatment. Results are mean values of at least four independent experiments performed in triplicate. For propidium iodide cell cycle analysis $5x10^5$ cells were fixed with EtOH 100% drop by drop while vortexing and stored o/n at 4ºC. To stain DNA content, cells were incubated for 30 min at 37ºC with 10 mg/ml RNase in PBS/Propidium iodide (Sigma-Aldrich) previous to analysis. Apoptosis was determined using Annexin V-FITC Apoptosis Detection kit (eBioscience, Thermo Fisher) with $4x10^5$ cells following instructions. Annexin V-FITC was incubated for 10 min at r.t. and propidium iodide staining previous to FACS analysis with BD FACSCantoTM II analyzer (BD Biosciences, San Jose, CA) was done to discriminate necrosis. Cell gate was done in singlet alive cells adjusting autofluorescence and analysis was performed after compensation. Acquired data analysis was performed using WinMDI 2.8 (Joe Trotter, The Scripps Institute, Flow Cytometry Core Facility) or Flowing Software v2.5.1 (Perttu Terho, University of Turku, Finland).

Migration Assays

Wound healing assays were performed in 6 well plates with 90% confluence. Cells were starved at 1% FBS o/n to minimize cell division during migration and 3 scratches/well with a tip were done. Phase contrast images were collected at indicated time points with a light microscope Olympus BX41 (Olympus, Hicksville, NY). Gap distance was measured using Image J software (https://imagej.nih.gov)

(NIH, Bethesda, MD) adjusting threshold back/white of sharpen finding edges. Assays using Transwell® chambers of 8 µm pore size (Cultek) were performed incubating the lower and upper inserts of the camber o/n with complete medium without FBS for indicated times. $1x10^5$ cells in starvation with 1% FBS o/n were seeded in the insert and cultivated for indicated periods of time. Inserts and wells were washed with PBS and fixed with 4% paraformaldehyde pH 7 for 20 min followed by staining with hematoxylin for 30 min and washing with distilled H_2O. After insert air-drying, cells were visualized under an upright Microscope and counted in 10 fields doing an average. Migrating cells were calculated by number of cells x 100 / number of cells seeded. At least 3 independent experiments were performed.

Plate soft agar and clonogenic cell survival assay

2D clonogenic assay was performed by culturing $1x10^3$ single cells in 6 well plates during 10-12 days. Plates were PBS-washed, and colonies were fixed with 4% formalin solution for 5 min, r.t. and stained with 0.5% Crystal Violet in MeOH for 1 h. Colonies of >25-50 cells were counted to obtain plating efficiency (nº of colonies counted*100/nº of cells plated). Colonies were photographed with an Olympus CX31 microscope and their numbers were counted using Image J in 8-bit format, adjusting the threshold to reduce non-specific background and counted by maxima as described previously (10). For 3D clonogenic tests, colonies were imagined with an Olympus CX31 microscope at 2, 3 and 6 wk. We counted colonies and measured colony diameter using Image J as described in clonogenic survival by analyzing particles. For soft agar assay protocol, 6 well plates with a low layer of 0.5% agar in

DMEM were prepared and kept at 4ºC for up 1 wk. $5x10^4$ cells were seeded in triplicate in a top layer of 0.4% agarose in DMEM with FBS. Cells were incubated at 37ºC in a humidified incubator for 10-50 d replacing complete media twice per wk. Colonies were stained with 0.005% Crystal Violet in MeOH for 90 min. For 3D clonogenic tests, colonies were imagined with an Olympus CX31 microscope at 2, 3 and 6 wk. Colonies were counted using Image J software.

RNA extraction of human CRC samples and NanosString gene expression profiling

An in-house NanoString panel (Suppl. Table S5) on the *n*Counter platform (NanoString Technologies) was used to interrogate gene expression on FFPE tissue, following the manufacturer's protocol. We used a customized gene-panel based on published pre-clinical and clinical research literature (11). Briefly, 10 µm-thick sections of formalin-fixed paraffin-embedded (FFPE) tumor tissues were examined by H&E. Only samples with ≥ 10% of tumor cellularity were processed for RNA purification (High Pure FFPET RNA isolation kit (Roche). When needed, macro-dissection was performed to enrich tumor cells and minimize stromal components. After excluding samples with suboptimal RNA integrity and content, the remaining samples were included in the nCounter analysis. The final set of data (n=115) was analyzed on the nSolver 4.0 Advanced Analysis module, using default settings to derive differentially expressed genes, pathway scores, and cell types scores. In Kaplan Meier survival plots, high and low *ZEB1* expression patient cohorts were established by using as cut off those in the third highest levels of expression

versus those in the two thirds lowest. Censored patients in Kaplan Meier plots indicated those that were withdrawn from the cohort before the final outcome is observed.

SUPPLEMENTARY TABLES

SUPPLEMENTARY TABLE S1: DEG upregulated and downregulated genes in LS174T and RKO with CTLKD and ZEB1KD

Top 25 upregulated and 25 downregulated DEG mRNA in LS174T^{ZEB1KD} vs LS174T^{CTLKD} ranked in descending level of significance

Gene symbol	logFC	AveExpr	P.Value	adj.P.Val
ME1	-1.44059361	0.19610181	1.45E-06	7.24E-03
NUP42	-1.28152004	3.99697802	1.90E-06	7.24E-03
MAL2	-1.26214817	2.74358118	4.39E-06	1.12E-02
GCSH	-1.19063185	4.42528995	4.26E-06	1.12E-02
H2BC5	1.4525145	2.53465262	9.57E-06	1.29E-02
CHML	-1.54513851	4.56332147	1.36E-05	1.48E-02
SOD3	1.57637756	-0.60309372	1.63E-05	1.65E-02
SCOC	-1.33910098	0.02143959	3.21E-05	2.12E-02
FOXJ1	2.29038481	-2.73328803	4.48E-05	2.47E-02
ZNF587B	-1.63300874	3.4489606	5.57E-05	2.54E-02
CNIH3	1.91958276	-1.0691996	5.99E-05	2.54E-02
PCDHGA1	-2.11793221	-1.18905044	7.89E-05	2.87E-02
CASTOR1	1.61944237	3.46192739	8.09E-05	2.87E-02
AC011511.4	-5.61074161	0.57201915	9.20E-05	2.97E-02
RNASE1	2.08790214	-0.09796149	9.83E-05	2.99E-02
TMEM160	1.5778436	2.72624262	1.18E-04	3.15E-02
PLA2G2A	1.62329446	-2.40005877	1.20E-04	3.15E-02
CYP1A1	2.05811033	3.32553393	1.44E-04	3.33E-02
KIF5C	-2.32048584	-3.06766268	1.52E-04	3.41E-02
PUS7L	-1.37004046	4.87993812	1.78E-04	3.57E-02
BX470111.1	-3.66346017	-1.20479609	2.07E-04	3.71E-02
NLRP2	-2.16062078	-3.17143363	2.28E-04	3.71E-02
PDP1	-1.19815091	6.16836697	2.18E-04	3.71E-02
INO80B	1.2117407	4.03251668	2.17E-04	3.71E-02
CTSA	1.23959893	6.89173587	2.10E-04	3.71E-02
AGT	1.532966	-2.93558385	2.26E-04	3.71E-02
ZNF578	2.51089293	-0.33726498	2.43E-04	3.75E-02
H2AC8	1.49246166	-0.09074971	2.99E-04	4.05E-02
PHOSPHO2	-1.51352997	0.43504403	3.14E-04	4.10E-02
ZBED8	-1.50851056	2.00289303	3.22E-04	4.13E-02
SLC16A6	-3.01025129	-1.82867677	3.87E-04	4.21E-02

CCDC85B	1.46001087	5.84605667	4.43E-04	4.26E-02
ZNF85	-2.42777004	-1.34240114	4.88E-04	4.30E-02
TRIM59	-1.20771892	-0.79756816	4.98E-04	4.30E-02
EPHA10	-1.83551457	-1.41518103	5.36E-04	4.31E-02
ZNF417	-1.46127517	3.15542819	5.34E-04	4.31E-02
ZNF117	-1.80290164	2.79586142	6.24E-04	4.37E-02
C19orf38	1.2019278	-1.45363097	6.52E-04	4.37E-02
LYZ	1.2970269	2.00846878	6.95E-04	4.37E-02
IGFBP6	1.34244804	4.74098262	6.57E-04	4.37E-02
AC002996.1	2.00218314	2.0225857	6.34E-04	4.37E-02
AC022414.1	3.22403053	-3.36844232	6.93E-04	4.37E-02
TPGS1	1.33933822	2.0309811	7.60E-04	4.53E-02
ZBTB26	-1.21133839	3.4303338	8.27E-04	4.55E-02
EMC10	1.19912302	6.05526028	8.19E-04	4.55E-02
SLC39A4	1.30524162	5.12268699	8.26E-04	4.55E-02
CCL15	1.46040186	-2.6429165	8.00E-04	4.55E-02
FXYD3	1.47213956	0.67295277	7.96E-04	4.55E-02
JAZF1	-2.79970102	0.27025852	8.65E-04	4.60E-02
ALG10B	-1.34769375	1.81395187	9.46E-04	4.72E-02

Top 25 upregulated and 25 downregulated DEG mRNA in RKO^{ZEB1KD} vs RKO^{CTLKD} ranked in descending level of significance

Gene Symbol	logFC	AveExpr	P.Value	adj.P.Val
PLAU	4.277567133	4.547482201	2.81E-09	1.59E-05
ITGA2	2.790041557	6.680326778	1.16E-08	4.44E-05
PKP3	2.888487962	5.851973616	1.61E-07	3.06E-04
PYGL	-1.425785455	5.337563652	3.08E-07	3.62E-04
ATP5MC2	-1.211726409	6.881397389	8.91E-07	6.97E-04
LSG1	-1.315726147	5.59470308	1.06E-06	7.72E-04
CDC42BPG	5.165246007	3.007421245	1.25E-06	8.66E-04
SH2D3A	4.256826934	3.246537857	1.32E-06	8.75E-04
ZNF165	3.954513816	3.365706161	1.68E-06	1.07E-03
EPHA1	5.29152613	1.768004492	1.84E-06	1.10E-03
CRB3	3.569444039	2.464720374	2.01E-06	1.10E-03
CCDC120	3.446060708	1.204389015	3.63E-06	1.82E-03
BSPRY	4.710909323	1.606117693	5.13E-06	2.30E-03
SMPDL3B	3.384893342	2.573482012	9.14E-06	3.88E-03
TMEM184A	3.43777413	0.278691444	1.06E-05	4.13E-03
PRRG2	3.078524874	2.015574376	1.16E-05	4.24E-03
TMC4	2.808941081	3.606774676	1.16E-05	4.24E-03

ZEB1	-1.977134026	3.682221217	1.17E-05	4.24E-03
AMOTL1	2.907350748	0.562410155	1.77E-05	5.62E-03
RINL	3.200621014	1.352723356	3.42E-05	8.03E-03
GET1-SH3BGR	-4.789259075	-1.128639191	3.27E-05	8.03E-03
SERPINA3	-3.352382758	-1.709196641	3.32E-05	8.03E-03
RASL10B	-1.131066971	-0.918930957	3.41E-05	8.03E-03
RIPK4	3.687302541	2.426812059	3.94E-05	8.59E-03
RAMAC	-1.204218509	4.357487204	4.17E-05	8.61E-03
C1orf210	3.865951924	1.100377319	5.73E-05	1.05E-02
PRKACB	-3.597450262	-0.564827465	6.07E-05	1.06E-02
OAF	-1.321093438	5.177233751	7.40E-05	1.21E-02
C2orf15	2.99564105	1.545254813	7.67E-05	1.24E-02
NSA2	-1.123459879	6.303951056	7.87E-05	1.26E-02
MPZL2	3.743549435	1.204550461	9.98E-05	1.52E-02
ZNF333	-1.778650006	3.142628832	1.09E-04	1.54E-02
ATG2A	-1.424981177	5.975397035	1.06E-04	1.54E-02
TFF2	-3.588530783	0.121816517	1.27E-04	1.72E-02
CPNE7	-1.436902929	4.801940862	1.38E-04	1.76E-02
TJP3	3.902071831	3.171939079	1.47E-04	1.84E-02
LYZ	-1.854348037	2.008468782	1.58E-04	1.92E-02
FAM151B	-1.438961368	-0.688477659	1.57E-04	1.92E-02
PMEPA1	-2.192202638	0.815389078	1.97E-04	2.26E-02
HSH2D	3.525567938	1.381534417	3.68E-04	3.45E-02
GJB3	3.881239898	1.326910728	3.89E-04	3.54E-02
SEC16B	-2.105867435	-0.9610908	4.53E-04	3.92E-02
QPRT	-2.464660235	1.543326389	4.56E-04	3.93E-02
UCN	-2.166189709	-0.673740418	4.94E-04	4.14E-02
TRPV3	3.108518571	-1.269955448	5.39E-04	4.32E-02
SLC9B1	-1.959713099	-0.853592569	5.42E-04	4.32E-02
PHTF2	-1.318327668	4.727287005	6.18E-04	4.64E-02
EHF	-2.506191156	2.652773522	6.66E-04	4.70E-02
FGD4	2.833198172	1.43231673	6.77E-04	4.76E-02

44 DEG mRNA in (RKO^{ZEB1KD} vs RKO^{CTLKD}) vs ($LS174T^{ZEB1KD}$ vs $LS174T^{CTLKD}$) ranked in descending level of significance

Gene symbol	log FC	P. value	adj.P.Val
EPHA1	5.03273	5.02E-06	6.38E-03
CDC42BPG	4.63733	7.57E-06	7.70E-03
BSPRY	4.59432	1.10E-05	9.22E-03
LCAL1	4.48077	1.17E-06	3.15E-03

ZNF165	4.43572	4.54E-06	6.38E-03
ZSCAN12P1	4.32813	4.22E-06	6.38E-03
AC010503.5	4.26995	7.79E-07	3.15E-03
PLAU	4.20568	8.31E-06	7.92E-03
AMOTL1	4.14241	1.59E-05	1.06E-02
SH2D3A	4.04400	6.37E-06	7.45E-03
C10orf55	3.71929	1.15E-04	4.41E-02
TMEM184A	3.54973	2.30E-05	1.40E-02
CCDC120	3.25225	1.59E-05	1.06E-02
CRB3	3.18648	1.47E-05	1.06E-02
HOOK1	2.83103	2.41E-05	1.41E-02
STYK1	2.78715	1.28E-04	4.66E-02
MAL2	2.73777	1.14E-04	4.41E-02
TMC4	2.62507	5.27E-05	2.59E-02
ITGA2	2.59052	1.24E-06	3.15E-03
SPINT2	2.54834	4.75E-08	7.25E-04
OR2A7	2.41523	6.84E-06	7.45E-03
EPCAM	2.38175	1.12E-06	3.15E-03
MPZL3	2.28756	2.73E-06	5.94E-03
PKP3	2.26892	3.42E-05	1.93E-02
ANKEF1	1.93464	1.70E-05	1.08E-02
ZNF33B	1.87667	1.28E-04	4.66E-02
PRSS22	1.74476	8.71E-05	3.80E-02
CHML	1.72215	3.57E-05	1.95E-02
ARHGEF5	1.69080	2.90E-07	2.21E-03
PATJ	1.64514	3.27E-06	6.23E-03
ARHGEF34P	1.60866	9.59E-06	8.60E-03
DMKN	1.58814	9.64E-05	4.06E-02
PICK1	1.32230	4.81E-05	2.45E-02
PLA2G12A	1.28323	9.84E-05	4.06E-02
BCAP29	1.21100	4.46E-05	2.34E-02
RAB27A	1.15647	1.39E-04	4.84E-02
LRRC8B	1.14780	7.69E-05	3.49E-02
MEST	1.09361	7.77E-05	3.49E-02
DDAH1	0.88784	1.16E-04	4.41E-02
ATP5MC2	-1.45923	4.70E-06	6.38E-03
QPRT	-2.94869	1.32E-04	4.68E-02
LYZ	-3.15137	1.15E-05	9.22E-03
TFF2	-4.23345	5.56E-05	2.65E-02
SERPINA3	-4.60475	1.31E-05	9.96E-03

<u>SUPPLEMENTARY TABLE S2</u>: EMT transcription factors and markers in LS174T and RKO with CTL and ZEB1KD

EMT transcription factors in LS174T and RKO with CTLKD and ZEB1KD in descending level of significance

LS174T^{ZEB1KD} vs LS174T^{CTLKD}

Gene symbol	Log FC	P. value
ZEB1	-1.54026617	0.00911728
SNAI3	0.26772993	0.71490668
SNAI1	0.19079734	0.73329829

RKO^{ZEB1KD} vs RKO^{CTLKD}

Gene symbol	Log FC	P. value
ZEB1	-1.0.3747464	0.00102783
SNAI3	0.59897782	0.44850933
SNAI1	0.35173264	0.52745689

EMT markers in LS174T and RKO with CTLKD and ZEB1KD in descending level of significance

(LS174T^{ZEB1KD} vs LS174T^{CTLKD}) vs (RKO^{ZEB1KD} vs RKO^{CTLKD})

Gene symbol	Log FC	P. value
CLDN1	-15.4709662	2.73e-99
TNC	16.4960837	4.69e-80
LAMA3	-15,99914	1,47e-78
CDH1	-15.6705538	7.59e-56
FNDC4	11.935194	4.40e-36
EPCAM	-3.4089737	1.16e-31
VIM	6,17253008	1.78e-15
TJP3	-6.07758244	1.85e-11
VEGFC	9.90554293	3.80e-11
LAMC2	-6,7798754	2,71e-08
COL4A4	-4.642	2.29e-07
MMP3	9.26214286	1.26e-06
OCLN	0.28402304	0.2950938

SUPPLEMENTARY TABLE S3: DEG GSEA pathways upregulated and downregulated in LS174T and RKO with CTLKD and ZEB1KD

Top 20 upregulated and 20 downregulated GSEA pathways in LS174T^{ZEB1KD} vs LS174T^{CTLKD} ranked in descending level of significance

GSEA pathway	pval	padj	ES	NES
Mitochondrial_translation_elongation	1.79E-06	6.57E-05	0.539076564	2.758794666
Mitochondrial_translation	1.79E-06	6.57E-05	0.51975055	2.672391711
Mitochondrial_protein_import	1.86E-06	6.57E-05	0.553956805	2.48927824
Antigen_processing-Cross_presentation	1.82E-06	6.57E-05	0.501205339	2.472910642
Degradation_of_the_extracellular_matrix	1.84E-06	6.57E-05	0.525750824	2.44830559
Neutrophil_degranulation	1.72E-06	6.57E-05	0.434443227	2.423007542
Regulation_of_RUNX2_expression_and_activity	1.83E-06	6.57E-05	0.49703533	2.409963126
Extracellular_matrix_organization	1.77E-06	6.57E-05	0.454613848	2.408777213
Arachidonic_acid_metabolism	1.90E-06	6.57E-05	0.617069374	2.407545348
The_citric_acid_(TCA)_cycle_and_respiratory_ electron_transport	1.80E-06	6.57E-05	0.471165103	2.401176535
Respiratory_electron_transport	1.84E-06	6.57E-05	0.512643832	2.400260851
Mitochondrial_translation_initiation	1.85E-06	6.57E-05	0.524470267	2.399627175
Ephrin_signaling	1.92E-06	6.57E-05	0.687535405	2.39712225
Heparan_sulfate/heparin_(HS-GAG)_metabolism	1.89E-06	6.57E-05	0.586425448	2.371717074
Mitochondrial_translation_termination	1.85E-06	6.57E-05	0.517906125	2.369594026
Respiratory_electron_transport,_ATP_synthesis_by_ chemiosmotic_coupling,_and_heat_production_by_ uncoupling_proteins	1.83E-06	6.57E-05	0.488726689	2.363452423
Formation_of_the_beta-catenin:TCF_transactivating_complex	1.85E-06	6.57E-05	0.516139847	2.340857312
Dectin-1_mediated_noncanonical_NF-kB_signaling	1.88E-06	6.57E-05	0.557294007	2.339063911
Translation	1.68E-06	6.57E-05	0.407096918	2.337745809
AUF1_(hnRNP_D0)_binds_and_destabilizes_mRNA	1.88E-06	6.57E-05	0.567927734	2.33747122
Mitotic_Prometaphase	2.28E-06	7.56E-05	-0.395085687	-2.110158466
Cilium_Assembly	2.28E-06	7.56E-05	-0.362545033	-1.938975662
Resolution_of_Sister_Chromatid_Cohesion	4.41E-06	1.26E-04	-0.410700281	-2.016404651
Amplification_of_signal_from_the_kinetochores	6.54E-06	1.59E-04	-0.441092599	-2.078198733
Amplification__of_signal_from_unattached__kinetochores _ via_a_MAD2__inhibitory_signal	6.54E-06	1.59E-04	-0.441092599	-2.078198733
Mitotic_Spindle_Checkpoint	6.59E-06	1.59E-04	-0.418565584	-2.033139654
Interactions_of_Rev_with_host_cellular_proteins	6.24E-05	8.33E-04	-0.452246012	-2.007999735
SUMOylation_of_DNA_replication_proteins	1.45E-04	1.61E-03	-0.41630671	-1.894774556
EML4_and_NUDC_in_mitotic_spindle_formation	1.62E-04	1.71E-03	-0.373867092	-1.809915724
Signaling_by_FGFR1_in_disease	2.47E-04	2.48E-03	-0.569860223	-2.056032286
Insulin_processing	3.49E-04	3.33E-03	-0.627440428	-2.065481039
RUNX1_regulates_transcription_of_genes_involved_in_ differentiation_of_keratinocytes	4.22E-04	3.98E-03	-0.809019909	-2.001527222

tRNA_processing	5.72E-04	5.11E-03	-0.349200071	-1.706990254
M_Phase	6.45E-04	5.62E-03	-0.247650696	-1.44162121
snRNP_Assembly	7.25E-04	6.16E-03	-0.439339084	-1.850285592
Metabolism_of_non-coding_RNA	7.25E-04	6.16E-03	-0.439339084	-1.850285592
RUNX3_regulates_RUNX1-mediated_transcription	1.00E-03	8.07E-03	-0.986716691	-1.528597227
Resolution_of_D-loop_Structures_through_Holliday_Junction_Intermediates	1.10E-03	8.57E-03	-0.508971043	-1.900893317
Nuclear_Pore_Complex_(NPC)_Disassembly	1.10E-03	8.57E-03	-0.49592609	-1.894464971
Anchoring_of_the_basal_body_to_the_plasma_membrane	1.10E-03	8.57E-03	-0.359258964	-1.702632149

Top 20 upregulated and 20 downregulated DEG GSEA pathways in RKO^{ZEB1KD} vs RKO^{CTLKD} ranked in descending level of significance

GSEA pathway	pval	padj	ES	NES
rRNA_processing	9.27E-06	1.73E-03	0.356942165	1.753814039
rRNA_processing_in_the_nucleus_and_cytosol	8.78E-06	1.73E-03	0.361934328	1.768726134
HDR_through_Homologous_Recombination_(HRR)_or_Single_Strand_Annealing_(SSA)	3.47E-05	4.32E-03	0.416824233	1.841551774
DNA_Repair	5.43E-05	5.59E-03	0.290475809	1.47296425
DNA_Double-Strand_Break_Repair	6.93E-05	6.37E-03	0.374917297	1.718653138
HDR_through_Homologous_Recombination_(HRR)	8.14E-05	6.70E-03	0.473433415	1.910010136
Homology_Directed_Repair	9.21E-05	7.29E-03	0.394315232	1.756331861
DNA_Replication	1.01E-04	7.39E-03	0.375840798	1.710066586
Interactions_of_Rev_with_host_cellular_proteins	1.14E-04	7.80E-03	0.462337398	1.8759537
RNA_Polymerase_I_Transcription_Initiation	1.30E-04	8.64E-03	0.468163463	1.87798679
Activation_of_the_pre-replicative_complex	1.78E-04	1.08E-02	0.600075981	2.052552069
S_Phase	2.32E-04	1.27E-02	0.337663496	1.587059411
Homologous_DNA_Pairing_and_Strand_Exchange	2.70E-04	1.39E-02	0.540137939	1.973483225
Major_pathway_of_rRNA_processing_in_the_nucleolus_and_cytosol	3.28E-04	1.61E-02	0.33100077	1.56358304
tRNA_processing	3.51E-04	1.68E-02	0.378805357	1.678070351
RNA_Polymerase_I_Transcription	3.71E-04	1.69E-02	0.396912457	1.712896653
Resolution_of_D-loop_Structures_through_Synthesis-Dependent_Strand_Annealing_(SDSA)	3.63E-04	1.69E-02	0.624220503	2.022947977
Synthesis_of_DNA	4.73E-04	2.05E-02	0.36267383	1.63621117
Resolution_of_D-Loop_Structures	4.77E-04	2.05E-02	0.563169359	1.956336459
RNA_Polymerase_I_Transcription_Termination	4.79E-04	2.05E-02	0.573936002	1.963140611
Degradation_of_the_extracellular_matrix	1.28E-06	5.46E-04	-0.605017546	-2.227482209
Transcriptional_regulation_of_pluripotent_stem_cells	1.59E-06	5.46E-04	-0.836160571	-2.172010546
Class_A/1_(Rhodopsin-like_receptors)	1.34E-06	5.46E-04	-0.604113078	-2.109148423
GPCR_ligand_binding	1.25E-06	5.46E-04	-0.522140266	-1.960024149
Extracellular_matrix_organization	1.13E-06	5.46E-04	-0.480357651	-1.945887721
Signaling_by_GPCR	1.06E-06	5.46E-04	-0.400030157	-1.687264301
GPCR_downstream_signalling	2.14E-06	6.30E-04	-0.397640474	-1.669011839

Formation_of_the_cornified_envelope	2.92E-06	7.50E-04	-0.682185983	-2.122890528
Keratinization	4.36E-06	9.96E-04	-0.675489468	-2.113704047
Nitric_oxide_stimulates_guanylate_cyclase	1.18E-05	2.02E-03	-0.896639065	-2.002448053
Cell_junction_organization	1.35E-05	2.13E-03	-0.56452546	-1.96155985
Biological_oxidations	2.32E-05	3.18E-03	-0.469236767	-1.799451915
G_alpha_(i)_signalling_events	2.19E-05	3.18E-03	-0.426135944	-1.706921919
Cell-cell_junction_organization	3.58E-05	4.32E-03	-0.616329527	-1.976847092
Non-integrin_membrane-ECM_interactions	4.23E-05	4.83E-03	-0.600590692	-1.967666459
cGMP_effects	5.12E-05	5.54E-03	-0.892279785	-1.92877225
Peptide_ligand-binding_receptors	5.99E-05	5.87E-03	-0.605015115	-1.949161665
TNFs_bind_their_physiological_receptors	7.42E-05	6.37E-03	-0.793766474	-1.993621618
Nuclear_Receptor_transcription_pathway	7.44E-05	6.37E-03	-0.63307391	-1.970058956
Phase_I_-_Functionalization_of_compounds	9.78E-05	7.39E-03	-0.547956756	-1.884767095

Top 20 upregulated and 20 downregulated DEG GSEA pathways in (RKO^{ZEB1KD} vs RKO^{CTLKD}) vs (LS174T^{ZEB1KD} vs LS174T^{CTLKD}) ranked in descending level of significance

GSEA pathway	pval	padj	ES	NES
Resolution_of_Sister_Chromatid_Cohesion	1.70E-06	1.18E-04	0.495058275	2.219730167
Mitotic_Prometaphase	1.62E-06	1.18E-04	0.447467283	2.169939931
Mitotic_Spindle_Checkpoint	1.70E-06	1.18E-04	0.488478208	2.168330087
M_Phase	1.50E-06	1.18E-04	0.346641729	1.820780136
Signaling_by_Rho_GTPases	1.49E-06	1.18E-04	0.312057555	1.644239016
Amplification_of_signal_from_the_kinetochores	3.45E-06	1.34E-04	0.501817569	2.16487538
Amplification__of_signal_from_unattached__ kinetochores_via_a_MAD2__inhibitory_signal	3.45E-06	1.34E-04	0.501817569	2.16487538
EML4_and_NUDC_in_mitotic_spindle_formation	3.41E-06	1.34E-04	0.46823666	2.072168873
RHO_GTPases_Activate_Formins	8.37E-06	3.19E-04	0.41732209	1.918021846
SUMOylation_of_DNA_replication_proteins	1.05E-05	3.85E-04	0.498671826	2.083491447
Insulin_processing	1.50E-05	5.40E-04	0.723483532	2.231080422
Interactions_of_Rev_with_host_cellular_proteins	3.17E-05	1.06E-03	0.489109673	1.996690676
Mitotic_Metaphase_and_Anaphase	3.65E-05	1.19E-03	0.341224563	1.695152178
Mitotic_Anaphase	4.61E-05	1.39E-03	0.338871902	1.682725539
Mitotic_Telophase/Cytokinesis	2.22E-04	4.80E-03	0.770037114	2.058028172
Cell_Cycle_Checkpoints	2.22E-04	4.80E-03	0.317456876	1.595637286
Signaling_by_MST1	3.89E-04	7.70E-03	0.941757692	1.783120385
RHO_GTPase_Effectors	5.34E-04	9.80E-03	0.297445272	1.519064704
Zinc_efflux_and_compartmentalization_by_the_ SLC30_family	6.68E-04	1.13E-02	0.933385256	1.767268047
SLC_transporter_disorders	7.69E-04	1.25E-02	0.453334024	1.811743168
Protein_localization	2.70E-06	1.18E-04	-0.354799457	-1.85569379

Transcriptional_regulation_by_RUNX2	2.54E-06	1.18E-04	-0.38989708	-1.931231416
Antigen_processing-Cross_presentation	2.49E-06	1.18E-04	-0.400238048	-1.93444378
Fatty_acid_metabolism	2.50E-06	1.18E-04	-0.421511988	-2.052963611
Regulation_of_RUNX2_expression_and_activity	2.46E-06	1.18E-04	-0.440338442	-2.091933636
Mitochondrial_protein_import	2.35E-06	1.18E-04	-0.507124792	-2.233815669
Mitochondrial_Fatty_Acid_Beta-Oxidation	2.20E-06	1.18E-04	-0.663638669	-2.392497021
Influenza_Infection	2.52E-06	1.18E-04	-0.53363207	-2.617903989
Ribosomal_scanning_and_start_codon_recognition	2.28E-06	1.18E-04	-0.645986508	-2.64831005
Mitochondrial_translation_termination	2.37E-06	1.18E-04	-0.596515147	-2.675080538
Mitochondrial_translation_initiation	2.37E-06	1.18E-04	-0.605433883	-2.715076735
Complex_I_biogenesis	2.28E-06	1.18E-04	-0.669105548	-2.722405652
Translation_initiation_complex_formation	2.28E-06	1.18E-04	-0.665809168	-2.729575759
Signaling_by_ROBO_receptors	2.63E-06	1.18E-04	-0.535749053	-2.749629004
Activation_of_the_mRNA_upon_binding_of_the_ cap-inding_complex_and_eIFs,_and_subsequent_ binding_to_43S	2.28E-06	1.18E-04	-0.671314836	-2.762998019
Influenza_Viral_RNA_Transcription_and_ Replication	2.48E-06	1.18E-04	-0.575807872	-2.766794991
Formation_of_the_ternary_complex,_and_subsequ ently,_the_43S_complex	2.26E-06	1.18E-04	-0.699872533	-2.78794804
rRNA_processing	2.71E-06	1.18E-04	-0.540941532	-2.839192088
rRNA_processing_in_the_nucleus_and_cytosol	2.68E-06	1.18E-04	-0.548204748	-2.859769895
The_citric_acid_(TCA)_cycle_and_respiratory_ electron_transport	2.55E-06	1.18E-04	-0.57648637	-2.878562053

<u>SUPPLEMENTARY TABLE S4</u>: Demographic and patient characteristics at baseline

Characteristics	*ZEB high (n=38)*	*ZEB low (n=77)*
Median age (range)-yr	60 (32-77)	67 (30-86)
>70 years of age-no. (%)	9 (24)	38 (49)
Male sex-no. (%)	14 (37)	43 (59)
ECOG performance status score of 0-1-no (%)	30 (79)	50 (66)
MSI-H-no. (%)*	4 (17)	13 (10)
Genotype-no.%**		
-*RAS* mutant	11 (29)	27 (35)
-*BRAF* mutant	17 (45)	21 (28)
-2WT	10 (26)	28 (37)
Primary location		
- Right side-no. %	18 (47)	32 (41)
Stage		
- Metachronous-no.%	- 16 (42)	- 24 (31)
Surgery primary tumor-no (%)	30 (79)	51 (66)
Presence of liver metastases-no (%)	15 (39)	49 (64)
Presence of peritoneal metastases-no (%)	16 (42)	27 (35)
No. organs affected-no.%		
-≥2 organs	11 (29)	37 (48)
ALP>ULN-no (%)***	15 (42)	35 (48)
LDH>ULN-no (%)****	11 (30)	37 (51)
CEA Mean (range)*****	77.4 (0.9-977)	637.5 (0.3-11363)

*3 patients with missing information; ** 1 patient with missing information; *** 6 patients with missing information;**** 7 patients with missing information;****5 patients with missing information

<u>Abbreviations</u>: ALP: alkaline phosphatase; CEA: Carcinoembryonic Antigen; ECOG: Eastern Cooperative Oncology Group; LDH: Lactate dehydrogenase; MSI-H: High Microsatellite instability; ULN: upper limit normality.

<u>SUPPLEMENTARY TABLE S5</u>: Gene signature used in RNA expression by Nanostring

Gene related family (BM2 vs BM1)	Number of genes	Evaluated genes
Immune-related	17	*CCL2, IL6, IL17, CCL5, CD8A, FASL, FAS, IL10, IL2, IFNG, TGFB1, ARG1, PD1, IL23, CXCL10, CXCL9, STAT1*
Sumoylation	7	*RANBP2, PIAS3, SAE, UCB9, PIAS2, PIAS1, PIAS4*
CAF	7	*S100A4, FAP, ACTA2, PDGFC, TGFB3, CXCL12, SMAD4*
EMT	6	*TWIST, ZEB1, ZEB2, SNAI1, SNAI2, BANCR*
BRAF-like signature	5	*VAV3, DUSP4, RNF43, CDX2, AXIN2*
Apoptosis	4	*BCL2, BCL2L1, BIRC5, MCL1*
Angiogenesis	3	*VEGFA, VEGFB, VEGFC*
Metalloproteinases	2	*MMP9, MMP7*
BRAF-cyclin activation	1	*RAC1*

<u>SUPPLEMENTARY TABLE S6</u>: 20 Genes upregulated/downregulated correlated with *ZEB1* in *BRAF* mutant CRC patients ranked in descending level of significance

Gene symbol	p	corr	Gene symbol	p	corr
ACTA2	1.94E-08	0.885654	*CCNO*	0.0013	-0.913837
SI2	2.63E-06	0.81138	*BAX*	0.0016	-0.817585
VEGFB	5.38E-06	0.79674	*TPI1*	0.0029	-0.770520
TGFB3	0.000156	0.708222	*IL32*	0.0038	-0.764086
MS4A4A	0.00036	0.857701	*NOS2*	0.0064	-0.735389
TGFB2	0.000768	0.832965	*CBLC*	0.0068	-0.731587
CSF1R	0.000983	0.823968	*COL17A1*	0.0088	-0.716112
NFIL3	0.00183	0.798888	*PARP4*	0.0184	-0.664606
ATM	0.00255	0.86769	*PDZK1IP1*	0.0220	-0.650345
ID4	0.00287	0.778242	*IRF1*	0.0293	-0.626560
DAB2	0.003	0.776125	*FBP1*	0.0302	-0.623719
ZEB2	0.00403	0.761193	*ENO1*	0.0323	-0.817838
KAT2B	0.00453	0.75493	*IFITM1*	0.0324	-0.617376
ITGA1	0.00472	0.752774	*DTX4*	0.0352	-0.609861
COL6A3	0.00475	0.752458	*TRIM21*	0.0367	-0.606176
TGFBR1	0.0059	0.740268	*CDX2*	0.0389	-0.433344
SIRPA	0.00855	0.717971	*BCL2L1*	0.0394	-0.599436
PECAM1	0.0088	0.716144	*PCK2*	0.0434	-0.590000
EPM2AIP1	0.00914	0.713752	*TNFS9*	0.0466	-0.583134
IFI16	0.00938	0.712022	*EIF4EBP1*	0.0475	-0.581096

<u>SUPPLEMENTARY TABLE S7</u>: *BRAF* mutant (V600E) demographic and patient characteristics at baseline

**Characteristics**	***ZEB* high (n=17)**	***ZEB* low (n=21)**
Median age (range)-yr	65 (42-77)	68 (40-84)
>70 years of age-no. (%)	8 (47)	11 (52)
Male sex-no. (%)	11 (65)	13 (62)
ECOG performance status score of 0-1-no (%)	12 (70)	12 (57)
MSI-H-no. (%)*	3 (27)	8 (42)
Primary location		
- Right side-no. %	10 (59)	14 (67)
Stage		
- Metachronous-no.%	- 8 (47)	- 6 (28)
Surgery primary tumor-no (%)	13 (76)	13 (62)
Presence of liver metastases-no (%)	7 (41)	10 (48)
Presence of peritoneal metastases-no (%)	7 (41)	12 (57)
No. organs affected		
-≥2 organs	7 (41)	11 (52)
ALP>ULN-no (%)	9 (53)	11 (52)
LDH>ULN-no (%)	4 (23)	9 (43)
CEA. Mean (range)	100.9 (3-977)	409.2 (0.3-7956)

*2 patients with missing information;

***RAS* mutant demographic and patient characteristics at baseline**

Characteristics	*ZEB* high (n=12)	*ZEB* low (n=23)
Median age (range)-yr	60 (40-75)	72 (35-84)
>70 years of age-no. (%)	1 (8)	14 (60)
Male sex-no. (%)	5 (42)	9 (39)
ECOG performance status score of 0-1-no (%)	9 (75)	13 (57)
MSI-H-no. (%)	0 (0)	3 (13)
Primary location		
- Right side-no. %	6 (50)	9 (39)
Stage		
- Metachronous-no.%	- 5 (42)	- 8 (35)
Surgery primary tumor-no (%)	10 (83)	16 (70)
Presence of liver metastases-no (%)	4 (33)	19 (83)
Presence of peritoneal metastases-no (%)	4 (33)	4 (17)
No. organs affected		
-≥2 organs	4 (33)	11 (48)
ALP>ULN-no (%)	3 (25)	10 (43)
LDH>ULN-no (%)	4 (33)	14 (61)
CEA. Mean (range)	38.62 (0.9-111.3)	635.13 (1.9-7176)

<u>Abbreviations</u>: ALP: alkaline phosphatase; CEA: Carcinoembryonic Antigen; ECOG: Eastern Cooperative Oncology Group; LDH: Lactate dehydrogenase; MSI-H: High Microsatellite instability; ULN: upper limit normality.

<u>SUPPLEMENTARY TABLE S8</u>: Type of therapy and activity in *BRAF* mutant patients

Variable	*ZEB* high (n=17)	*ZEB* low (n=21)
Type of therapy		
- Surgery-no (%)	8	1
- Systemic therapy-no (%)	9	16
- Best supportive care-no (%)	0	4
Overall response		
- Partial response%	12	61
- Stable disease%	62	31
- Progressive disease%	26	8
Median progression free survival (95% CI)	9.2 (5-13.4)	7.1 (3.4-10.9)
Median overall survival (95% CI)	21.4 (6.3-36.4)	7.9 (5-10.7)

Abbreviation: CI: confidence interval.

<u>SUPPLEMENTARY TABLE S9</u>: Univariate analysis of overall survival and progression free survival in *BRAF* mutant CRC patients

Overall survival	B	SE	Wald	df	Sig	Exp(B)	95% CI for Exp (B)	
							Lower	Upper
Sex	0.024	0.415	0.003	1	0.953	1.025	0.455	2.309
ZEB1 higher than mean	-0.988	0.474	4.344	1	0.037	0.372	0.147	0.943
Stage	0.505	0.433	1.362	1	0.243	1.657	0.710	3.868
LDH higher than ULN	0.940	0.422	4.957	1	0.026	2.560	1.119	5.856
ALP higher than ULN	-0.177	0.410	0.185	1	0.667	0.838	0.375	1.873
LEU >11.000	0.849	0.486	3.057	1	0.080	2.338	0.902	6.059
M1 location	-0.029	0.203	0.020	1	0.887	0.972	0.653	1.446
Age of diagnosis	0.030	0.022	1.911	1	0.167	1.030	0.988	1.075

Progression free survival	B	SE	Wald	df	Sig	Exp(B)	95% CI for Exp (B)	
							Lower	Upper
Sex	0.490	0.356	1.900	1	0.168	1.633	0.813	3.278
ZEB1 higher than mean	-0.581	0.374	2.410	1	0.121	0.560	0.269	1.165
Stage	1.019	0.398	6.552	1	0.010	2.770	1.270	6.044
LDH higher than ULN	0.477	0.378	1.596	1	0.206	1.611	0.769	3.377
ALP higher than ULN	0.458	0.368	1.551	1	0.213	1.581	0.769	3.252
LEU >11.000	0.781	0.441	3.137	1	0.077	2.184	0.920	5.183
M1 location	-0.030	0.188	0.025	1	0.875	0.971	0.671	1.404
Age of diagnosis	0.021	0.017	1.502	1	0.220	1.022	0.987	1.057

<u>Abbreviations</u>: ALP: alkaline phosphatase; LDH: Lactate dehydrogenase; LEU: leukocytes per microliter; M1: metastasis; ULN: upper limit normality.

<u>SUPPLEMENTARY TABLE S10</u>: DNA primers used for genotyping by PCR

Mouse Gene	Forward 5'to 3'	Reverse 5' to 3'
Braf $^{LSL-V600E}$	TGAGTATTTTTGTGGCAACTG C	CTCTGCTGGGAAAGCGGC (mut / wt)
Kras $^{LSL-G12D}$	GTCGACAAGCTCATGCGGG	CCATGGCTTGAGTAAGTCTGC (mut)<br>CGCAGACTGTAGAGCAGCG (wt)
Vil1 ^{Cre}	GCCTTCTCCTCTAGGCTCGT	AGGCAAATTTTGGTGTACGG (mut)<br>TATAGGGCAGAGCTGGAGGA (wt)
Zeb1	CCAGAGCCCCAGCACTATTCT CC	TCACACGGCAAACGACTGTCCTG (mut)<br>ACCGCACCTGGTTTACGACACTC (wt)

SUPPLEMENTARY TABLE S11: shRNA lentiviral constructs

Human Genes	Sequence (5' to 3')	Source	Catalog Reference
shCtl	Not available (proprietary)	Santa Cruz Biotechnology	sc108080V
sh*ZEB1*	GAAGCAGGAUGUACAGUAA<br>GGCGAUAGAUGGUAAUGUA<br>CCAGAACAGUGUUUAUUCU	Santa Cruz Biotechnology	sc38643V

<u>SUPPLEMENTARY TABLE S12</u>: siRNA oligonucleotides

Human Genes	Sequence 5' to 3'	Reference
siCtl	GGUUACGAACUAAGCUAUA	12
si*BRAF*	AACAGUCUACAAGGGAAAGUG	13
si*KRAS*	CAGGGUGUUGAUGAUGCCUUCUAUA	14
si*ZEB1*	AACUGAACCUGUGGAUUAUTT	12

<u>SUPPLEMENTARY TABLE S13</u>: Primary Antibodies

Primary antibodies used in immunohistochemistry / immunofluorescence

Antibody	Producer	Catalogue	Source	IHQ dilution
β-catenin E247	Abcam	ab32572	Rabbit	1:200
CK20 E9	SCBT	sc-271183	Mouse	1:50
Cleaved Caspase 3 (Asp175)	Cell Signaling	9661	Rabbit	1:200
E-cadherin clone 36	BD	BD 610181	Mouse	1:50
Fibronectin EP5	SCBT	sc-8422	Mouse	1:50
KI67	Biorbyt	Orb247042	Rabbit	1:200
Lysozyme EPR2994(2)	Abcam	ab108508	Rabbit	1:1000
Occludin E-5	SCBT	sc-133256	Mouse	1:50
pAKT1/2/3 B-5	SCBT	sc-271966	Rabbit	1:2000
pERK-1/2 E4	SCBT	sc-7383	Mouse	1:200
Vimentin RV202	SCBT	sc-32322	Mouse	1:50
ZEB1 H102	SCBT	sc-25388	Rabbit	1:50
ZEB1	Sigma Aldrich	HAP027524	Rabbit	1:100

Primary antibodies used in Western Blot

Antibody	Producer	Catalogue	Source	WB dilution
α-tubulin B-5-1-2	Sigma Aldrich	T6074	Mouse	1:500
AKT1 C-20	SCBT	sc-1618	Goat	1:500
ERK1 C-16	SCBT	sc-93	Rabbit	1:500
GAPDH 14C10	Cell Signaling	2118L	Rabbit	1:1000
KRAS	Proteintech	12063-1-AP	Rabbit	1:500
pAKT1/2/3 B5	SCBT	sc-271966	Rabbit	1:500
pERK E4	SCBT	sc-7383	Mouse	1:200
BRAF H145	SCBT	sc-9002	Rabbit	1:500
ZEB1 H102	SCBT	sc-25388	Rabbit	1:400
ZEB1	Sigma Aldrich	HPA027524	Rabbit	1:500

<u>SUPPLEMENTARY TABLE S14</u>: DNA primers used for determination of gene expression by qRT-PCR

Human Gene	Reference	Forward 5' to 3'	Reverse 5' to 3'
ADAM17	15	CTGTGGTGCAAAAGCAGAAA	TGCCAAATGCCTCATATTCA
ARHGAP4	This study	CTGCGCTTTGACTACCACC	CAGTGTCGCCTTCAGAGTCT
BABAM1	This study	AGCCCCTGAGGTCCAAATTC	GAGCCGTTGAACGACTCCAG
BRAF	16	AGAAAGCACTGATGATGAGAG G	GGAAATATCAGTGTCCCAACCA
CDH1	17	TGCTGCAGGTCTCCTCTTGG	AGTCCCAGGCGTAGACCAAG
CDC25A	18	AGAGTCAACTAATCCAGAGAAG G	AGCAACTGTATGAAAGAGATAA CC
CDH1	19	TGAAGGTGACAGAGCCTCTGG AT	TGGGTGAATTCGGGCTTGTT
CENPF	20	ACGGAAGGTACTGAGTTTGAG CCA	GAGTTGCCATGGTTGTTCTTCG CA
CLDN1	21	GTCTTGACTCCTTGCTGAATCT G	CACCTCATCGTCTTCCAAGCAC
GAPDH	22	CGACTTCAACAGCAACTCCCAC TCTTCC	TGGGTGGTCCAGGGTTTCTTAC TCCTT
DHRS2	This study	ATGGGAATGAGTCTCTCTGGAA	CGTTGACGTAGCTGGCATC
DICER1	23	AAAATTGTCCATCATGTCCTCG C	CCACCAGGTCAGTTGCAGTT
DSC2	24	CGGAGATTGTTGCGGTTGA	GGAAAGACGTGCTGCTGTATCA
EHF	25	CAGTGCAGTAGTGACCTGTTC	CTGTGCTACCATAGTTGGTGTC
FAM3C	This study	TGAAGGCCATACAAGATGGAA C	CACAGAAGACCCAGTTGTCTCT A
FGD4	26	TCTCATCAGTCGCTTTGAAGGA	GGGTTCTAGGAGCATTTAGGTT C
FNDC4	27	TGGTCATCATTGTGGTGGTGTT	AGAGTCCTCAGGGAAGGCCA
GAPDH	28	TGCACCACCAACTGCTTAGC	GGCATGGACTGTGGTCATGAG
HOOK1	29	CAGACATTCAATACTGCCTCAC C	CCCCAACATCCTCTTTAATTCGG
KLK10	30	ACTGCGGAAACAAGCCACT	CCCTGGTGGTACTTGGGAT
KRAS	31	GGGGAGGGCTTTCTTTGTGTA	GTCCTGAGCCTGTTTTGTGTC
MDM2	32	GGATCCTTTGCAAGCGCCAC	TCAAAGGACAGGGACCTGCG
OCLN	33	TGGGACAGAGGCTATGGAAC	ATGCCCAGGATAGCACTCAC
PRDX3	34	ACAGCCGTTGTCAATGGAGAG	ACGTCGTGAAATTCGTTAGCTT
PYCARD	This study	GATCCAGGCCCCTCCTCAG	GCATCTTGCTTGGGTTGGTG
SNAI1	35	TTCCAGCAGCCCTACGACCAG	GCCTTTCCCACTGTCCTCATC
TFF2	36	TCGTCCTGGGGCTATGTGC	TGAGACCTCCATGACGCACTG
TICAM2	This study	CTCTTGGGGTAAAAGGCACA	TGTTGGCCCCTCTGTTGTAT
TJP3	37	GCTTCCTCAAGGGCAAGAGCA T	CGTGTCAGGTTCTGGAATGGCA
TMPRSS2	This study	GCAGTGGTTTCTTTACGCTGT	CCGCAAATGCCGTCCAATG
TWIST1	38	CGACGAGCTGGACTCCAAG	CCTCCATCCTCCAGACCGA
VIM	33	TCTGGATTCACTCCCTCTGG	CGTGATGCTGAGAAGTTTCG

ZEB1	39	AACTGCTGGCAAGACAAC	TTGCTGCAGAAATTCTTCCA
ZEB1	40	AGCAGTGAAAGAGAAGGGAATGC	GGTCCTCTTCAGGTGCCTCAG
ZEB2	This study	GAAAAGCAGTTCCCTTCTGC	GCCTTGAGTGCTCGATAAGG

<u>SUPPLEMENTARY TABLE S15</u>: Analysis of BM subtype, BOR and survival of published studies of treated *BRAF* mutant mCRC patients

Reference	*BRAF* patients (n)	Stage	Therapy	%BM 1/BM2	BOR BM1/BM2	Survival BM1/BM2	Transcriptomic characteristics BM1	Transcriptomic characteristics BM2
41	218	I-IV	CHT	31/69	NR	BM1<BM2	EMT/Immune	G2M, E2F targets, glycolysis
42	46	IV	CHT	52/48	NR	BM1=BM2	Immune	WNT
This study	41	IV	CHT	NR	NR	ZEB1$_H$>ZEB$_L$	ZEB1$_H$/EMT$_L$	ZEB1$_L$/EMT$_H$
43	47	IV	Triplet*	32/68	38/7	BM1>BM2	Immune	E2F, G2M
44	460	IV	Triplet/ Doublet**	32/68	33.3/14.9 19.8/19.6	NR	Immune MAPK	WNT, glycolysis, OXPHOS, MYC, DNA repair

* Triplet therapy (dabrafenib, trametinib and panitumumab)
** Triplet therapy (encorafenib, binimetinib and cetuximab) and doublet therapy (encorafenib and cetuximab)

<u>SUPPLEMENTARY TABLE S16</u>: P values of the Figures

Figure	Comparison	Statistical test	P	Significance
**Fig 1A**	KVZ+/+ vs KVZ-/-	Log-rank	0.0438	*
	BVZ+/+ vs BVZ+/-	Log-rank	0.0086	**
**Fig 1B**	colon KVZ+/+ vs KVZ+/- at 4.5m	2-tailed t-test	0.0001	***
	colon KVZ+/+ vs KVZ+/- at 8m		0.0335	*
	colon BVZ+/+ vs BVZ+/- at 4.5m	2-tailed t-test	0.1483	ns
	colon BVZ+/+ vs BVZ+/- at 8m		<0.0001	***
**Fig 1D**	Weight Z+/- vs KVZ+/+	Tukey	<0.0001	***
	Weight KVZ+/+ vs KVZ+/-		0.0868	ns
	Weight Z1+/- vs KVZ+/-		0.0004	***
	Weight Z+/- vs BVZ+/+		<0.0001	***
	Weight BVZ+/+ vs BVZ+/-		0.0758	ns
	Weight Z+/+ vs BVZ+/-		<0.0001	***
**Fig 1E**	Liver weigh KVZ+/+ vs KVZ+/-	Tukey	0.0993	ns
	Liver weight KVZ+/+ vs Z+/-		0.0259	*
	Liver weight KVZ+/- vs Z+/-		0.8842	ns
	Lung weight KVZ+/+ vs KVZ+/-		0.3387	ns
	Lung weight KVZ+/+ vs Z+/-		0.1562	ns
	Lung weight KVZ+/- vs Z+/-		0.8076	ns
**Fig 1F**	Liver weight BVZ+/- high vs BVZ+/+ high	Tukey	0.0002	***
	Liver weight BVZ+/- low vs BVZ+/- high		<0.0001	***
	Liver weight BVZ+/- low vs BVZ+/+ low		0.7779	ns
	Liver weight BVZ+/+ low vs BVZ+/+ high		0.0167	*
	Liver weight BVZ+/+ low vs Z+/-		0.5725	ns
	Liver weight BVZ+/+ high vs Z+/-		0.2416	ns
	Liver weight BVZ+/- low vs Z+/-		0.9953	ns
	Liver weight BVZ+/- high vs Z+/-		<0.0001	***
**Fig 1G**	Lung weight BVZ+/- high vs BVZ+/+ high	Tukey	0.9902	ns
	Lung weight BVZ+/- low vs BVZ+/- high		0.0002	***
	Lung weight BVZ+/- low vs BVZ+/+ low		0.8597	ns
	Lung weight BVZ+/+ low vs BVZ+/+ high		0.0007	***
	Lung weight BVZ+/+ low vs Z+/-		0.9888	ns
	Lung weight BVZ+/+ high vs Z+/-		0.0030	**
	Lung weight BVZ+/- low vs Z+/-		0.6755	ns
	Lung weight BVZ+/- high vs Z+/-		0.0022	**
**Fig 2B**	Colon KI67 KVZ+/+ vs KVZ+/-	MW	0.014	*

	Colon β-catenin KVZ+/+ vs KVZ+/-	MW	0.152	ns
	Colon Cleaved Caspase 3 KVZ+/+ vs KVZ+/-	MW	0.1	ns
	Colon ZEB1 KVZ+/+ vs KVZ+/-	MW	0.0159	*
	Colon Alcian Blue KVZ+/+ vs KVZ+/-	MW	0.0019	**
	Colon KI67 BVZ+/+ vs BVZ+/-	MW	0.0244	*
	Colon β-catenin BVZ+/+ vs BVZ+/-	MW	0.0012	**
	Colon Cleaved Caspase 3 BVZ+/+ vs BVZ+/-	MW	0.8329	ns
	Colon ZEB1 BVZ+/+ vs BVZ+/-	MW	0.0043	**
	Colon Alcian Blue BVZ+/+ vs BVZ+/-	MW	<0.0001	***
	SI KI67 KVZ+/+ vs KVZ+/-	MW	0.0091	**
	SI β-catenin KVZ+/+ vs KVZ+/-	MW	0.0114	*
	SI Lysozyme KVZ+/+ vs KVZ+/-	MW	0.029	*
	SI ZEB1 KVZ+/+ vs KVZ+/-	MW	0.0031	**
	SI Alcian Blue KVZ+/+ vs KVZ+/-	MW	0.7813	ns
	SI KI67 BVZ+/+ vs BVZ+/-	MW	<0.0001	***
	SI β-catenin BVZ+/+ vs BVZ+/-	MW	0.0379	*
	SI Lysozyme BVZ+/+ vs BVZ+/-	MW	0.0273	*
	SI ZEB1 BVZ+/+ vs BVZ+/-	MW	0.0003	***
	SI Alcian Blue BVZ+/+ vs BVZ+/-	MW	0.0769	ns
**Fig 3A**	C pAKT BVZ+/+ vs BVZ+/-	MW	0.0012	**
	C pERK BVZ+/+ vs BVZ+/-	MW	0.0008	***
	SI pAKT BVZ+/+ vs BVZ+/-	MW	0.0017	**
	SI pERK BVZ+/+ vs BVZ+/-	MW	0.0027	**
**Fig 3B**	C E-cadherin KVZ+/+ vs KVZ+/-	t-test	0.0033	**
	C Vimentin KVZ+/+ vs KVZ+/-	t-test	0.8571	ns
	SI E-cadherin KVZ+/+ vs KVZ+/-	t-test	0.0002	***
	SI Vimentin KVZ+/+ vs KVZ+/-	t-test	0.9307	ns
	C E-cadherin BVZ+/+ vs BVZ+/-	t-test	0.0317	*
	C Vimentin BVZ+/+ vs BVZ+/-	t-test	0.0007	***
	SI E-cadherin BVZ+/+ vs BVZ+/-	t-test	0.0036	**
	SI Vimentin BVZ+/+ vs BVZ+/-	t-test	0.7857	ns
**Fig 3C**	C E-cadherin KVZ+/+ vs KVZ+/-	t-test	0.0002	***
	C Occludin KVZ+/+ vs KVZ+/-	t-test	0.012	*
	C Vimentin KVZ+/+ vs KVZ+/-	t-test	0.0002	***
	C Fibronectin KVZ+/+ vs KVZ+/-	t-test	0.0014	**
	C E-cadherin BVZ+/+ vs BVZ+/-	t-test	0.0114	*
	C Occludin BVZ+/+ vs BVZ+/-	t-test	0.0049	**
	C Vimentin BVZ+/+ vs BVZ+/-	t-test	<0.0001	***

	C Fibronectin BVZ+/+ vs BVZ+/-	t-test	0.0053	**
**Fig 4B**	LS174T CTL vs KRASKD	Dunnett	<0.0001	***
	LS174T CTL vs ZEB1KD		<0.0001	***
	RKO CTL vs BRAFKD	Dunnett	0.0059	**
	RKO CTL vs ZEB1KD		<0.0001	***
**Fig 4F**	*ZEB1* in ZEB1KD vs CTL in LS174T	t-test	0.0003	***
	ZEB2 in ZEB1KD vs CTL in LS174T	t-test	0.7107	ns
	SNAI1 in ZEB1KD vs CTL in LS174T	t-test	0.0564	ns
	TWIST in ZEB1KD vs CTL in LS174T	t-test	0.1538	ns
	ZEB1 in ZEB1KD vs CTL in RKO	t-test	<0.0001	***
	ZEB2 in ZEB1KD vs CTL in RKO	t-test	0.763	ns
	SNAI1 in ZEB1KD vs CTL in RKO	t-test	0.3716	ns
	TWIST in ZEB1KD vs CTL in RKO	t-test	0.8422	ns
**Fig 5A**	*KRAS* mut CTL vs ZEB1 KD 24h	Bonferroni	<0.0001	***
	KRAS mut CTL vs ZEB1 KD 48h		<0.0001	***
	KRAS mut CTL vs ZEB1 KD 72h		<0.0001	***
	BRAF mut CTL vs ZEB1 KD 24h	Bonferroni	>0.9999	ns
	BRAF mut CTL vs ZEB1 KD 48h		>0.9999	ns
	BRAF mut CTL vs ZEB1 KD 72h		0.0114	**
**Fig 5B**	*KRAS* mut G0 CTL vs G0 Z1KD	t-test	0.0439	*
	KRAS mut G1 CTL vs G1 Z1KD	t-test	0.36	ns
	KRAS mut S CTL vs S Z1KD	t-test	0.3425	ns
	KRAS mut G2/M CTL vs G2/M Z1KD	t-test	0.0082	**
	BRAF mut G0 CTL vs G0 Z1KD	t-test	0.1062	ns
	BRAF mut G1 CTL vs G1 Z1KD	t-test	0.0011	**
	BRAF mut S CTL vs S Z1KD	t-test	0.1219	ns
	BRAF mut G2/M CTL vs G2/M Z1KD	t-test	0.0046	**
**Fig 5C**	*KRAS* MUT % Annex CTL vs ZEB1KD	t-test	0.0276	*
	BRAF MUT % Annex CTL vs ZEB1KD	t-test	0.7204	ns
**Fig 5D**	*KRAS* 2D platting eff % Annex CTL vs ZEB1KD	t-test	0.0078	**
	BRAF 2D platting eff % Annex CTL vs ZEB1KD	t-test	0.2602	ns
**Fig 5E**	*KRAS* 3D platting eff % Annex CTL vs ZEB1KD	t-test	0.045	*
	BRAF 3D platting eff % Annex CTL vs ZEB1KD	t-test	0.0444	*
**Fig 5F**	*KRAS* mut wound area CTL vs ZEB1KD 24h	Bonferroni	>0.9999	ns
	KRAS mut wound area CTL vs ZEB1KD 48h		<0.0001	***
	KRAS mut wound area CTL vs ZEB1KD 96h		<0.0001	***
	BRAF mut wound area CTL vs ZEB1KD 6h	Bonferroni	0.5209	ns
	BRAF mut wound area CTL vs ZEB1KD 24h		0.3132	ns

	BRAF mut wound area CTL vs ZEB1KD 48h		0.9309	ns
**Fig 5G**	*KRAS* mut % migrating cells CTL vs ZEB1KD	t-test	<0.001	***
	BRAF mut % migrating cells CTL vs ZEB1KD	t-test	0.2503	ns
**Fig 5H**	Mice LS174T CTL vs ZEB1KD	2-way ANOVA	0.0003	***
	Ex vivo Mice LS174T CTL vs ZEB1KD	t-test	0.0147	*
**Fig 5I**	Mice RKO CTL vs ZEB1 KD	2-way ANOVA	<0.0001	***
	Ex vivo Mice RKO CTL vs ZEB1 KD	t-test	0.036	*
**Fig 6E**	*KRAS* mut ZEB1KD vs CTL *FAM3C*	t-test	0.0036	**
	KRAS mut KRASKD vs CTL *FAM3C*	t-test	0.0053	**
	KRAS mut ZEB1KD vs CTL *KLK10*	t-test	0.0089	**
	KRAS mut KRASKD vs CTL *KLK10*	t-test	<0.0001	***
	KRAS mut ZEB1KD vs CTL *DHRS2*	t-test	0.0164	*
	KRAS mut KRASKD vs CTL *DHRS2*	t-test	<0.0001	***
	KRAS mut ZEB1KD vs CTL *PRDX3*	t-test	0.0075	**
	KRAS mut KRASKD vs CTL *PRDX3*	t-test	0.0004	***
	KRAS mut ZEB1KD vs CTL *BABAM1*	t-test	0.0059	**
	KRAS mut KRASKD vs CTL *BABAM1*	t-test	<0.0001	***
	KRAS mut ZEB1KD vs CTL *ADAM17*	t-test	0.0138	*
	KRAS mut KRASKD vs CTL *ADAM17*	t-test	0.0006	***
	KRAS mut ZEB1KD vs CTL *MDM2*	t-test	0.0027	**
	KRAS mut KRASKD vs CTL *MDM2*	t-test	0.0066	**
	KRAS mut ZEB1KD vs CTL *CEINPF*	t-test	0.0047	**
	KRAS mut KRASKD vs CTL *CEINPF*	t-test	<0.0001	***
	KRAS mut ZEB1KD vs CTL *DICER1*	t-test	0.0026	**
	KRAS mut KRASKD vs CTL *DICER1*	t-test	0.0084	**
	KRAS mut ZEB1KD vs CTL *TICAM2*	t-test	0.0003	***
	KRAS mut KRASKD vs CTL *TICAM2*	t-test	0.0049	**
	KRAS mut ZEB1KD vs CTL *CDC25A*	t-test	0.03	*
	KRAS mut KRASKD vs CTL *CDC25A*	t-test	0.0204	*
	BRAF mut ZEB1KD vs CTL *FAM3C*	t-test	<0.0001	***
	BRAF mut BRAFKD vs CTL *FAM3C*	t-test	0.0233	*
	BRAF mut ZEB1KD vs CTL *KLK10*	t-test	<0.0001	***
	BRAF mut BRAFKD vs CTL *KLK10*	t-test	0.001	***
	BRAF mut ZEB1KD vs CTL *DHRS2*	t-test	0.0003	***
	BRAF mut BRAFKD vs CTL *DHRS2*	t-test	0.001	***
	BRAF mut ZEB1KD vs CTL *PRDX3*	t-test	0.0006	***
	BRAF mut BRAFKD vs CTL *PRDX3*	t-test	0.0025	**
	BRAF mut ZEB1KD vs CTL *BABAM1*	t-test	<0.0001	***

	BRAF mut BRAFKD vs CTL *BABAM1*	t-test	0.001	***
	BRAF mut ZEB1KD vs CTL *DSC2*	t-test	0.0002	***
	BRAF mut BRAFKD vs CTL *DSC2*	t-test	0.0102	*
	BRAF mut ZEB1KD vs CTL *TFF2*	t-test	0.0293	*
	BRAF mut BRAFKD vs CTL *TFF2*	t-test	0.0135	*
	BRAF mut ZEB1KD vs CTL *HOOK1*	t-test	0.0236	*
	BRAF mut BRAFKD vs CTL *HOOK1*	t-test	0.0251	*
	BRAF mut ZEB1KD vs CTL *ARHGAP4*	t-test	0.0004	***
	BRAF mut BRAFKD vs CTL *ARHGAP4*	t-test	0.0217	*
	BRAF mut ZEB1KD vs CTL *TMPRSS2*	t-test	0.0152	*
	BRAF mut BRAFKD vs CTL *TMPRSS2*	t-test	0.0311	*
	BRAF mut ZEB1KD vs CTL *FGD4*	t-test	0.0352	*
	BRAF mut BRAFKD vs CTL *FGD4*	t-test	0.0031	**
	BRAF mut ZEB1KD vs CTL *PYCARD*	t-test	<0.0001	***
	BRAF mut BRAFKD vs CTL *PYCARD*	t-test	0.0046	**
	BRAF mut ZEB1KD vs CTL *EHF*	t-test	0.0063	**
	BRAF mut BRAFKD vs CTL *EHF*	t-test	0.0055	**
**Fig 6F**	*BRAF* mut CRC CTL vs ZEB1KD EMT signature	t-test	0.1	ns
**Fig 6G**	*CDH1* ZEB1 KD vs CTL KD in LS174T	t-test	0.416	ns
	TJP3 ZEB1KD vs CTL KD in LS174T	t-test	0.5072	ns
	OCLN ZEB1 KD vs CTL KD in LS174T	t-test	0.0144	*
	CLDN1 ZEB1 KD vs CTL KD in LS174T	t-test	0.0425	*
	VIM ZEB1KD vs CTL KD in LS174T	t-test	0.0182	*
	FNDC4 ZEB1 KD vs CTL KD in LS174T	t-test	0.8801	ns
	CDH1 ZEB1 KD vs CTL KD in RKO	t-test	0.0176	*
	TJP3 ZEB1KD vs CTL KD in RKO	t-test	0.1598	ns
	OCLN ZEB1 KD vs CTL KD in RKO	t-test	0.3724	ns
	CLDN1 ZEB1 KD vs CTL KD in RKO	t-test	0.0202	*
	VIM ZEB1KD vs CTL KD in RKO	t-test	0.0194	*
	FNDC4 ZEB1 KD vs CTL KD in RKO	t-test	0.0206	*
**Fig 7D**	PFS *ZEB1* low versus *ZEB1* high	Log rank	0.0513	ns
**Fig 7E**	OS *ZEB1* low versus *ZEB1* high	Log-rank	0.0405	*

SUPPLEMENTARY REFERENCES

1. Dankort D, et al. A new mouse model to explore the initiation, progression and therapy of BRAFV600E-induced lung tumors. *Genes Dev.* 2007; 21(4):379-384.

2. Jackson EL, et al. Analysis of lung tumor initiation and progression using conditional expression of oncogenic K-ras. *Genes Dev*. 2001;15(24):3243-3248.

3. Madison BB, et al. Cis Elements of the Villin Gene Control expression in restricted domains of the vertical (crypt) and horizontal (duodenum, cecum) axes of the intestine. *J Biol Chem*. 2022;277(36):33275-33283.

4. Takagi T, et al. δEF1, a zinc-finger and homeodomain transcription factor, is required for skeleton patterning in multiple lineages. *Development*. 1998;125(1):21-31.

5. Li B, Dewey CN. RSEM: accurate transcript quantification from RNA-Seq data with or without a reference genome. *BMC Bioinformatics*. 2011;12:323.

6. Law CW, et al. voom: Precision weights unlock linear model analysis tools for RNA-seq read counts. *Genome Biol*. 2014;15(2):R29.

7. Korotkevich G, et al. Fast gene set enrichment analysis [preprint]. https://doi.org/10.1101/060012. Posted on *bioRxiv* February 01, 2021.

8. Yu G, et al. clusterProfiler: an R package for comparing biological themes among gene clusters. *OMICS*. 2012;16(5):284-287.

9. Barbie DA, et al. Systematic RNA interference reveals that oncogenic KRAS-driven cancers require TKB1. *Nature*. 2009;462(7269):108-112.

10. Rafehi H, et al. Clonogenic assay: adherent cells. *J Vis Exp.* 2011;13(49):2573.

11. Pedrosa L, et al. The tumor microenvironment in colorectal cancer therapy. *Cancers*. 2019;11(8):1172.

12. Sánchez-Tilló E, et al. ZEB1 Promotes invasiveness of colorectal carcinoma cells through the opposing regulation of uPA and PAI-1. *Clin Cancer Res*. 2013;19(5):1071-1082.

13. Calleros L, et al. Oncogenic Ras, but not (V600E)B-RAF, protects from cholesterol depletion-induced apoptosis through the PI3K/AKT pathway in colorectal cancer cells. *Carcinogenesis*. 2009;30(10):1670-1677.

14. Al-Ghamdi S., et al. Cten is targeted by Kras signalling to regulate cell motility in the colon and pancreas. *PLoS One*. 2011;6(6):e20919.

15. Fu YR, et al. MicroRNA miR-21 attenuates human cytomegalovirus replication in neural cells by targeting Cdc25a. *J Virol.* 2015;89(2):1070-1082.

16. Bailey AM, et al. FXR silencing in human colon cancer by DNA methylation and KRAS signaling. *Am J Physiology Gastrointest Liver Physiol.* 2014;306(1):G48-58.

17. Dobin A, et al. STAR: ultrafast universal RNA-seq aligner. *Bioinformatics*. 2013;29(1):15-21.

18. Dulloo I, et al. Hypoxia-inducible TAp73 supports tumorigenesis by regulating the angiogenic transcriptome. *Nat Cell Biol.* 2015;17(4):511-523.

19. Ding X, et al. MiR-137-3p Inhibits Colorectal Cancer Cell Migration by Regulating a KDM1A-Dependent Epithelial–Mesenchymal Transition. *Dig Dis Sci*. 2021;66(7):2272-2282.

20. Yang S, et al. BMP-6 promotes E-cadherin expression through repressing δEF1 in breast cancer cells. *BMC Cancer*. 2007;7:211-224.

21. Tu Y, et al. Upregulation of hsa-miR-31-3p induced by ultraviolet affects keratinocytes permeability barrier by targeting CLDN1. *Biochem Biophys Res Commun*. 2020;532(4):626-632.

22. Peña C, et al. E-cadherin and vitamin D receptor regulation by SNAIL and ZEB1 in colon cancer: Clinicopathological correlations. *Hum Mol Genet.* 2005;14(22):3361-3370.

23. Khan K, et al. Desmocollin switching in colorectal cancer. *Br J Cancer*. 2006;95(10):1367-1370.

24. Zhang Y, et al. Frequent transcriptional inactivation of Kallikrein 10 gene by CpG island hypermethylation in non-small cell lung cancer. C*ancer Sci*. 2010;101(4):934-940.

25. Cheng J, et al Circ_0007624 suppresses the development of esophageal squamous cell carcinoma *via* targeting miR-224-5p/CPEB3 to inactivate the EGFR/PI3K/AKT signaling. *Cell Signal*. 2022;99:110448.

26. Dai X, et al. Circular RNA circFGD4 suppresses gastric cancer progression via modulating miR-532-3p/APC/β-catenin signalling pathway. *Clin Sci (Lond)*. 2020;134(13):1821-1839.

27. Frühbeck G, et al. FNDC4, a novel adipokine that reduces lipogenesis and promotes fat browning in human visceral adipocytes. *Metabolism*. 2020;108:154261.

28. Gilliver SC, et al. Sex dimorphism in wound healing: the roles of sex steroids and macrophage migration inhibitory factor. *Endocrinology*. 2008;149(11):5747-57.

29. Liu K, et al. Regulation of p53 by TopBP1: a Potential Mechanism for p53 Inactivation in Cancer. *Mol Cell Biol*. 2009;29(10):2673-2693.

30. Saini JS, et al. Nicotinamide Ameliorates Disease Phenotypes in a Human iPSC Model of Age-Related Macular Degeneration. C*ell Stem Cell*. 2017;20(5):635-647.

31. de Freitas A, et al. Identification of TLT2 as an engulfment receptor for apoptotic cells. *J Immunol*. 2012;188(12):6381-6388.

32. Choi HS, et al. Identification of hepatotoxicity related genes induced by toxaphene in HepG2 cells. *Mol Cell Toxicol*. 2011;7(1):53-60.

33. Kim MS, et al. MEST induces Twist-1-mediated EMT through STAT3 activation in breast cancerMEST induces Twist-1-mediated EMT through STAT3 activation in breast cancers. *Cell Death Differ*. 2019;26(12):2594-2606.

34. Yingnan Y. (2010-08-20) Functional Studies of Y-Box Binding protein 1 in breast cancer. ScholarBank@NUS Repository.

35. Guaita S, et al Snail Induction of Epithelial to Mesenchymal Transition in Tumor Cells Is Accompanied by *MUC1* Repression and *ZEB1* Expression. *J Biol Chem*.. 2002;227(42):39209-39216.

36. Liu J, et al. The regulation of trefoil factor 2 expression by the transcription factor Sp3. *Biochem Biophys Res Comm* 2012;427(2):410-414.

37. Saygili E, et al. A drug-responsive multicellular human spheroid model to recapitulate drug-induced pulmonary fibrosis. *Biomed Mater*. 2022;17(4):5021.

38. Takeshi K, et al. Generation of human hepatic progenitor cells with regenerative and metabolic capacities from primary hepatocytes. *eLife*. 2019;8:e47313.

39. Shuai L, et al. Hepato-specific micro RNA-122 facilitates accumulation of newly synthesized miRNA through regulating PRKRA. *Nucleic Acids Res*. 2012;40(2):884-891.

40. Zhang GJ, et al. High expression of ZEB1 correlates with liver metastasis and poor prognosis in colorectal cancer. *Oncol Lett*. 2013;5(2):564-568.

41. Barras D, et al. BRAFV600E mutant colorectal cancer subtypes based on gene expression. *Clin Cancer Res*. 2017;23(1):104-115.

42. Moretto R, et al. Exploring clinical and gene expression markers of benefit from FOLFOXIRI/bevacizumab in patients with BRAF-mutated metastatic colorectal cancer: Subgroup analysis of the TRIBE2 study. *Eur J Cancer*. 2021;153:16-26.

43. Middleton G, et al. *BRAF*-Mutant Transcriptional subtypes predict outcome of combined BRAF, MEK, and EGFR blockade with dabrafenib, trametinib, and panitumumab in patients with Colorectal Cancer. *Clin Cancer Res*. 2020;26(11): 2466-2476.

44. Kopetz S, et al. Encorafenib, Binimetinib, and Cetuximab in BRAF V600E-Mutated Colorectal Cancer. *N Engl J Med*. 2019;381:1632-1643.

LEGENDS TO SUPPLEMENTARY FIGURES

SUPPLEMENTARY FIGURE S1

(A) Individual stainings of Fig. 3C. Scale bar 20µm. (B) Lysates from LS174Tand RKO cells incubated with PD98059, LY294002 or with their solvent (Untr) were immunoblotted for pERK1/2, ERK, pAKT and GAPDH. (C) Reduced expression of ZEB1 by siRNA. Lysates from LS174T and RKO cells interfered with siCtl (*CTL*) or siZEB1 (*ZEB1KD*) were blotted for ZEB1 and GAPDH. (D) Reduced expression of ZEB1 by lentiviral transduction. Lysates from LS174T and RKO cells stably interfered with a control lentivirus (CTL) or against *ZEB1* (*ZEB1KD*) were blotted for ZEB1 and GAPDH or α-tubulin. (E) Heatmap of leading edge genes involved in the enrichment score of EMT process in *BRAF* mut CRC in presence or absence of *ZEB1* (n=3), corresponding to Fig. 6F. At least three independent experiments were done.

SUPPLEMENTARY FIGURE S2

(A) Heatmap of EMT transcription factors in *BRAF* mut CRC patients of Fig. 7B.

Supplementary Figure 1

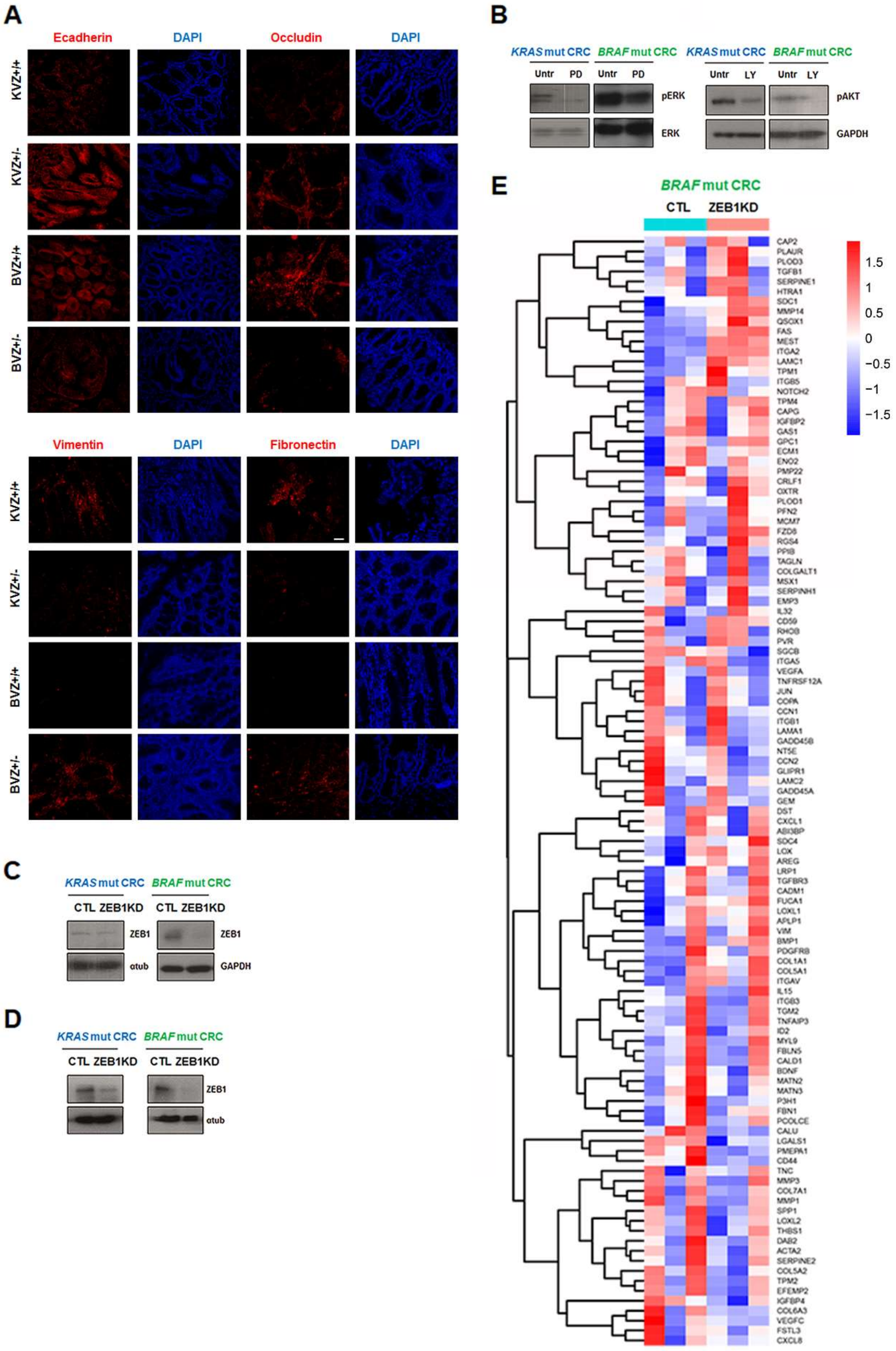

Supplementary Figure 2

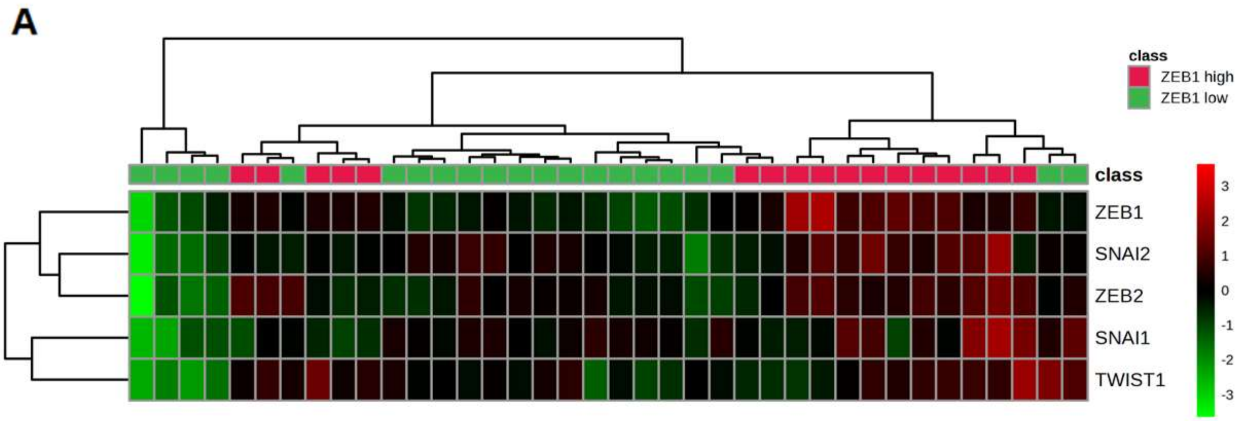

Supplement: Supplemental data [file jciinsight-8-164629-s197.pdf]
